# Succinylation-related molecular activities in cancer: metabolic adaptations, immune landscape, and prognostic significance in colorectal cancer

**DOI:** 10.3389/fimmu.2025.1571446

**Published:** 2025-05-20

**Authors:** Zhihang Jiang, Xiaoqing Li, Long Hu, Zheng Jiang

**Affiliations:** ^1^ Department of Gastroenterology, The First Affiliated Hospital of Chongqing Medical University, Chongqing, China; ^2^ Chongqing Key Laboratory of Molecular Oncology and Epigenetics, The First Affiliated Hospital of Chongqing Medical University, Chongqing, China; ^3^ XJTLU Wisdom Lake Academy of Pharmacy, Xi’an Jiaotong-Liverpool University, Suzhou, China

**Keywords:** succinylation, tumor immune microenvironment, prognostic model, colorectal cancer, PCED1A

## Abstract

**Background:**

Succinylation, a key post-translational modification, plays a crucial role in metabolic regulation and tumor progression. However, its influence on the tumor immune microenvironment and its prognostic implications remain unclear. A systematic pan-cancer analysis of succinylation-related molecular activities is needed.

**Methods:**

Bulk transcriptomic, single-cell RNA sequencing, and spatial transcriptomic data across pan-cancer from TCGA, GEO, TISCH, and multiple other databases were analyzed. Succinylation scores were calculated using Gene Set Variation Analysis (GSVA). The interactions between succinylation scores, immune infiltration, tumor microenvironment, tumor mutational burden, and immunotherapy response were assessed. A succinylation-based prognostic model was constructed and validated in colorectal cancer (CRC) cohorts. PCED1A protein expression was evaluated by immunohistochemistry and Western blotting. The function of PCED1A in CRC was investigated through *in vitro* experiments.

**Results:**

Succinylation scores were significantly altered in multiple tumor types. Higher succinylation scores correlated with mitochondrial oxidative phosphorylation, while lower succinylation scores were linked to immune cell differentiation. Spatial transcriptomic analysis showed a negative correlation between succinylation scores and immune cell activity in tumor-adjacent regions. A prognostic model consisting of 11 succinylation-related genes (ATP6V1C2, CAPS, DAPK1, P4HA1, PCED1A, RASL10B, AGT, EREG, HYAL1, SARAF, and SLC4A4) was developed. High-risk patients exhibited significantly shorter overall survival. PCED1A was upregulated in CRC and positively associated with SIRT5. Overexpression of PCED1A promoted intracellular protein desuccinylation, along with enhanced CRC cell proliferation, migration, and invasion.

**Conclusion:**

Our analysis demonstrates that succinylation-related molecular activities display distinct expression patterns across cancers, which are associated with metabolic regulation, immune modulation, and disease prognosis. The succinylation-based prognostic model provides a novel risk stratification tool for CRC, while PCED1A-dependent succinylation regulation may serve as a potential therapeutic target.

## 1 Introduction

Cancer is a multifaceted and complex cellular disease that progresses through intricate histological and molecular biological stages, which makes it one of the most pressing public health challenges worldwide. Advances in high-throughput sequencing (HTS) and bioinformatics technologies have revolutionized cancer research, and offered unprecedented insights into the genomic landscape of tumors (1). Advancements in technology have allowed scientists to identify millions of genomic alterations, which play potential roles in tumorigenesis and progression. However, the interaction between these genomic changes and the tumor microenvironment remains insufficiently understood, particularly regarding their immunological relevance and influence on immunotherapy responses. Additionally, the pronounced genetic and molecular heterogeneity observed across cancer types and within individual tumors underscores the importance of conducting pan-cancer studies. Such comprehensive analyses are essential for identifying novel biomarkers and therapeutic targets, which can lay the foundation for personalized therapeutic strategies and improve patient outcomes. Colorectal cancer (CRC), which ranks among the most common and aggressive malignancies globally, continues to pose significant challenges despite substantial advancements in treatment, including surgery, chemotherapy, and targeted therapies. The prognosis of CRC strongly depends on the stage at diagnosis, with patients diagnosed at a localized stage achieving a five-year survival rate exceeding 90%, whereas those presenting with distant metastases face a survival rate of less than 10% (2). Therefore, identifying novel molecular markers for CRC is crucial to improving the poor prognosis and treatment outcomes of CRC patients. Additionally, as a “cold tumor” with limited responsiveness to immunotherapy, it is highly desirable to discover valuable target genes that can improve the tumor immune microenvironment of CRC. Such discoveries could enhance the recognition of tumor antigens and increase the activity of tumor-infiltrating immune cells, thereby enabling a greater number of CRC patients to benefit from immunotherapy.

Under normal physiological conditions, cells primarily rely on mitochondrial oxidative phosphorylation (OXPHOS), which provides energy for cellular processes. However, most cancer cells undergo metabolic reprogramming, which shifts their energy production to aerobic glycolysis and results in the accumulation of succinate—a hallmark metabolic alteration critical for tumor adaptation (3). Succinate is not only a critical intermediate in the tricarboxylic acid cycle but also regulates cellular functions through succinylation, an epigenetic modification. Succinylation is a reversible acylation modification occurring on lysine residues, which is dynamically regulated by intracellular levels of succinate and succinyl-CoA (4). High levels of succinylation have been shown to reprogram the pentose phosphate pathway in gastric cancer, which provides energy and metabolic intermediates to support cell proliferation (5). Studies indicate that succinylation is widely distributed across various metabolic enzymes and transcriptional regulators, and it plays critical roles in metabolic pathways such as the tricarboxylic acid cycle, fatty acid metabolism, and oxidative phosphorylation (6). Furthermore, succinylation modifies chromatin-associated proteins, which alters the transcriptional state of genes and regulates the expression of tumor-related genes (7). For instance, lysine acetyltransferase 2A (KAT2A)-mediated histone H3 lysine 79 (H3K79) succinylation upregulates the expression of tyrosine 3-monooxygenase/tryptophan 5-monooxygenase activation protein zeta (YWHAZ), which encodes 14-3-3ζ, thereby enhancing the migratory and invasive capabilities of tumor cells (8). Sirtuin 5 (SIRT5), one of the most extensively studied desuccinylases, regulates protein activity by removing succinyl groups, thereby affecting cellular metabolic balance (6). SIRT5 modulates the succinylation status of various target proteins and exhibits complex roles across different tumor types. In hepatocellular carcinoma (HCC), SIRT5 deficiency leads to increased succinylation and activity of Acyl-CoA Oxidase 1 (ACOX1), which is directly associated with oxidative stress and DNA damage responses in HCC. These findings suggest that SIRT5 plays a protective role in liver function and inhibits the progression of HCC (9). In CRC, SIRT5 activates mitochondrial malic enzyme 2 (ME2) by desuccinylating lysine 346, which enables cancer cells to sustain mitochondrial respiration under glutamine-deficient conditions. This metabolic adaptation supports energy production and promotes CRC cell proliferation and tumorigenesis (10). Within the tumor microenvironment, succinylation not only regulates metabolic and signaling pathways but also closely influences immune cell functionality. SIRT5 deficiency leads to excessive succinylation of PKM2, which increases IL-1β production, thereby altering the polarization of tumor-associated macrophages (TAMs) and fostering an immunosuppressive environment (11). As a dynamically regulated epigenetic modification, succinylation plays a crucial role in cancer metabolic reprogramming and tumor microenvironment modulation. Elucidating the molecular mechanisms of succinylation and its functions in cancer will help uncover novel targets for tumor metabolic regulation and provide a theoretical foundation for the development of innovative anticancer therapies.

In this study, we systematically analyzed the expression profiles of succinylation-related genes across pan-cancer datasets and established molecular subtypes based on succinylation scores using unsupervised clustering. GSVA was used to compute succinylation scores in single-cell and spatial transcriptomic data, we further investigated the heterogeneity of succinylation scores in the TME and their relationship with intercellular communication. The results demonstrated that cells with high succinylation scores in CRC were significantly associated with mitochondrial oxidative phosphorylation and the electron transport chain, whereas cells with low succinylation scores were closely linked to immune cell differentiation. Spatial transcriptomic analysis revealed a negative correlation between succinylation scores and immune cell activity in tumor-adjacent regions. Additionally, through survival analysis of TCGA and independent validation cohorts, we developed a succinylation-related prognostic model comprising 11 core genes for risk stratification in CRC patients. Then, we validated the expression levels of the key gene PCED1A via immunohistochemistry. Functional experiments using CRC cell lines demonstrated that high PCED1A expression significantly promoted CRC cell proliferation, migration, and invasion, and enhanced intracellular protein desuccinylation. This study integrated bioinformatics analyses and experimental validations to elucidate the potential prognostic and therapeutic value of succinylation-related genes in CRC, which provides novel targets and directions for personalized therapeutic strategies.

## 2 Materials and methods

### 2.1 Data collection

Among the top 30 succinylation-related genes ranked by relevance score in the GeneCards database, 29 protein-coding genes were selected for further analysis. Somatic mutation data (mutation annotation format), RNA-seq data (STAR-Counts), and clinical information for 33 cancer types (9938 samples) were obtained from the Cancer Genome Atlas (TCGA) via the R package “TCGAbiolink” (v2.28.4). The gene expression matrix for 30 tissue types (7788 samples) and corresponding metadata from the Genotype-Tissue Expression (GTEx) project were sourced from UCSC Xena (http://xena.ucsc.edu/public/). Immunotherapy-related expression profiles were accessed using the following dataset identifiers: KIRC_GSE67501, NSCLC_GSE135222, STAD_PRJEB25780, SKCM_PRJEB23709, SKCM_Nathanson2017, SKCM_GSE91061, SKCM_GSE115821, and GBM_PRJNA482620. Additionally, gene expression and survival data for two independent CRC cohorts were retrieved from GSE39582 and GSE17536.

Single-cell transcriptome data were obtained from the Gene Expression Omnibus (GEO) (https://www.ncbi.nlm.nih.gov/geo/) and the Tumor Immune Single-cell Hub (TISCH) (http://tisch.comp-genomics.org/home/). The single-cell RNA-seq datasets covered multiple cancer types, including CRC (GSE166555, 66,050 cells), BRCA (GSE161529, 332,168 cells), KIRC (GSE159115, 27,669 cells), STAD (GSE134520, 41,554 cells), OV (EMTAB8107, 32,386 cells), PAAD (CRA001160, 57,443 cells), PRAD (GSE176031, 18,807 cells), ESCA (GSE160269, 208,658 cells), CHOL (GSE138709, 33,990 cells), and NSCLC (GSE117570, 11,453 cells). Additionally, immunotherapy-related single-cell RNA-seq datasets included SKCM (GSE120575, 16,291 cells), SCC (GSE123813, 25,891 cells), BLCA (GSE145281, 14,474 cells), and BCC (GSE123813, 52,884 cells).

Spatial transcriptome data for CRC were retrieved from Human Colorectal Cancer: Whole Transcriptome Analysis (https://www.10xgenomics.com/).

### 2.2 Succinylation score development

The “gsva” function from the “GSVA” package was used with the following parameters: (1) method = “ssgsea”, (2) kcdf = “Poisson”, and (3) min.sz = 10, to compute normalized enrichment scores (succinylation scores) for succinylation-related genes in individual samples. Tumor samples were stratified into high- and low-Succinylation groups based on the median succinylation score. The same Gene Set Variation Analysis (GSVA) approach was applied to single-cell RNA-seq and spatial transcriptomic datasets to determine succinylation scores at the single-cell or spatial resolution.

To assess the robustness of the succinylation score, GSVA scores were computed for C6 oncogenic signature gene sets from the Molecular Signatures Database (MSigDB) across all TCGA samples, followed by Spearman correlation analysis between succinylation scores and these GSVA scores.

### 2.3 Single-cell transcriptome analysis

The selection criteria for scRNA-seq datasets were defined as follows: (1) solid tumors, (2) inclusion of both tumor and corresponding normal samples, (3) sample size ≥5 with a total cell count exceeding 10,000, and (4) comprehensive representation of conventional tumor microenvironment components, including malignant/epithelial cells, stromal cells, and immune cells.

For each dataset, the MAESTRO pipeline was applied to perform quality control, batch effect correction, clustering, and cell-type annotation. Cell quality control criteria included (1) nFeatures >200 and (2) a mitochondrial gene ratio <20%. Data integration was achieved by mitigating confounding factors using the R package “harmony”, followed by data normalization, cell clustering, and visualization. Cell types were annotated based on established marker genes.

Differentially expressed genes (DEGs) between high- and low-Succinylation groups in cancer and immune cells were identified separately using the “FindMarkers” function with default parameters. Significant DEGs (adjusted *p*-value <0.05 and average fold-change >1) were then selected for Gene Set Enrichment Analysis (GSEA). Cell–cell paracrine communication analysis was conducted using the R package “CellChat” with the count matrix as input, and significant interaction pairs were identified using the Wilcoxon rank-sum test (*P*-value <0.05).

### 2.4 Analysis of spatial transcriptome data

Spatial transcriptome data were processed using the R package “Seurat” (12). The workflow included normalizing unique molecular identifier (UMI) counts, scaling the data, and identifying highly variable features via “SCTransform” (13). Principal component analysis (PCA) was then applied for dimensionality reduction and unsupervised clustering using the “RunPCA” function. Default parameters were used, with clustering based on the top 30 principal components. Additionally, the “SpatialFeaturePlot” function was utilized for subgroup identification and gene expression visualization.

### 2.5 Investigation of succinylation-related immune characteristics at the pan-cancer level

Firstly, the absolute abundances of 22 immune cell types in TCGA samples were inferred using the “CIBERSORT” algorithm, with the gene expression matrix (FPKM matrix) and “LM22” signature as references. Furthermore, differences in immune checkpoint and immunomodulation-related gene expression were compared to evaluate the potential clinical efficacy of immunotherapy across different succinylation groups.

### 2.6 Genomic variation analysis

The “maftools” R package was used to generate a waterfall plot illustrating the distribution of genes with high somatic mutation frequencies in patients at the pan-cancer level. Copy number variation (CNV) data were obtained from TCGA, and patients in different subtypes were analyzed using the “gistic2” module of the GenePattern website (14).

### 2.7 Survival analysis

To evaluate succinylation-related survival across 33 TCGA cancer types, Kaplan–Meier survival curves were generated to estimate survival differences between the two groups. Statistical significance was determined using the log-rank test. Survival analyses were performed using the R packages “survival” and “survminer”.

### 2.8 Analysis of succinylation signature-based classifications in CRC

Two patient clusters were identified in TCGA-COAD using the unsupervised consensus clustering algorithm implemented in the R package “ConsensusClusterPlus” (15), with 1000 iterations to ensure classification stability. The optimal number of clusters was determined based on the relative change in the area under the cumulative distribution function (CDF) curve.

### 2.9 Construction of CRC-succinylation predictor

To identify optimal prognostic genes, least absolute shrinkage and selection operator (Lasso) regression was performed using the “glmnet” package, CoxBoost analysis using the “CoxBoost” package, and stepwise Akaike information criterion (stepAIC) selection using the “MASS” package. These methods were applied to determine the most contributory succinylation-related genes associated with colon cancer prognosis from DEGs specific to CRC subtypes. The identified prognostic genes with the highest predictive value were then incorporated into a multivariate Cox proportional hazards regression model:

A succinylation-based scoring system was developed as a linear combination of the regression coefficients derived from the multivariate Cox model, weighted by the normalized expression levels of prognostic genes. Patients were stratified into high- and low-risk groups based on the median risk score calculated by this system. Differences in overall survival (OS) between these groups were assessed using Kaplan–Meier survival analysis and log-rank statistical tests. Finally, external validation was conducted in independent cohorts to confirm the robustness of the model.

### 2.10 Immunohistochemistry

Twenty pairs of paraffin-embedded sections, including tumor tissues and their matched adjacent normal tissues, were collected from patients who underwent radical CRC surgery at the Department of Pathology, The First Affiliated Hospital of Chongqing Medical University, between September 2021 and March 2022. This study was approved by the Ethics Committee of The First Affiliated Hospital of Chongqing Medical University (No. 2023–222).

The sections were baked and fully dewaxed in xylene, followed by rehydration through a graded ethanol series. Antigen retrieval was performed by heating the sections in sodium citrate buffer at 95–98°C for 15 minutes. After blocking, the sections were incubated overnight at 4°C with the primary antibody (“Anti-PCED1A,” 1:100, CUSABIO, CSB-PA867128LA01HU). The next day, they were incubated with a secondary antibody and developed using DAB (3,3’-diaminobenzidine) chromogen.

### 2.11 Western blotting

Total protein was extracted from CRC cells using RIPA buffer and quantified with a BCA Protein Assay Kit (Beyotime, China). Protein samples were separated by SDS-PAGE and transferred onto PVDF membranes. The membranes were blocked with 5% non-fat milk, washed with TBST, and incubated overnight at 4°C with primary antibodies. The next day, they were incubated with secondary antibodies (1:10,000, EarthOx, e030120 and e030110) for 2 hours. Protein signals were detected using ECL reagents (Beyotime, China).

The primary antibodies used were as follows: anti-PCED1A (1:800, CUSABIO, CSB-PA867128LA01HU), anti-SIRT5 (1:1000, Immunoway, YN5806), anti-β-actin (1:10,000, Affinity, T0022), and anti-succinyllysine (1:1000, PTM Biolabs, PTM-401).

### 2.12 Cell culture and transfection

The CRC cell lines SW480 and HCT116 were obtained from the American Type Culture Collection (ATCC) and cultured in Dulbecco’s Modified Eagle Medium (DMEM) supplemented with 10% fetal bovine serum (FBS) and 1% penicillin-streptomycin. Cells were maintained at 37°C in a humidified incubator with 5% CO_2_. PCED1A overexpression was induced by transfecting the cells with a PCED1A expression plasmid using HighGene Plus Transfection Reagent (ABclonal, China) following the manufacturer’s protocol. PCED1A knockdown was achieved by transfecting cells with two short hairpin RNA (shRNA) constructs using the same transfection reagent. The shRNA sequences were as follows: sh-PCED1A-1 (5′–3′: CAGAAAGACTCACTGCTCACATTCAAGAGATGTGAGCAGTGAGTCTTTCTG) and sh-PCED1A-2 (5′–3′: CTACTTCCTCACTCGTGTTTATTCAAGAGATAAACACGAGTGAGGAAGTAG).

### 2.13 Cell proliferation assay and transwell assay

For the cell proliferation assay, 10 μL of Cell Counting Kit-8 reagent was added to each well at 24-hour intervals, and absorbance at 450nm was measured using a microplate reader to assess cell viability. Cell migration and invasion abilities were evaluated using Transwell chambers (8.0μm pore size, Corning, US). For the invasion assay, the upper surface of the membrane was pre-coated with 50 μL of diluted Matrigel (1:8 dilution in serum-free medium) and incubated at 37°C for 1 hour. Cells were serum-starved overnight, then resuspended in 200 μL of serum-free medium and seeded into the upper chamber at a density of 1 × 10^5^ cells per well. The lower chamber was filled with 600 μL of medium containing 20% FBS. After 24 hours of incubation at 37°C, non-invading cells on the upper surface were removed with a cotton swab. The invading cells on the lower surface were fixed with 4% paraformaldehyde for 15 minutes, stained with 0.1% crystal violet for 20 minutes, and photographed under a microscope (5 random fields per well). Migration assays were conducted using the same procedure, except that the upper chamber was not coated with Matrigel.

### 2.14 Statistical analysis

All statistical analyses were conducted using R software (v4.3.1). The Wilcoxon test was applied for pairwise comparisons between two groups (* *p* < 0.05; ** *p* < 0.01; *** *p* < 0.001; **** *p* < 0.0001). Survival analysis was performed using the Kaplan–Meier method and the log-rank test. The *p*-value < 0.05 was considered statistically significant. Correlation coefficients were evaluated using Spearman correlation analysis.

## 3 Results

### 3.1 Characterization of succinylation-related molecular activities across cancer types

**Figure 1:**
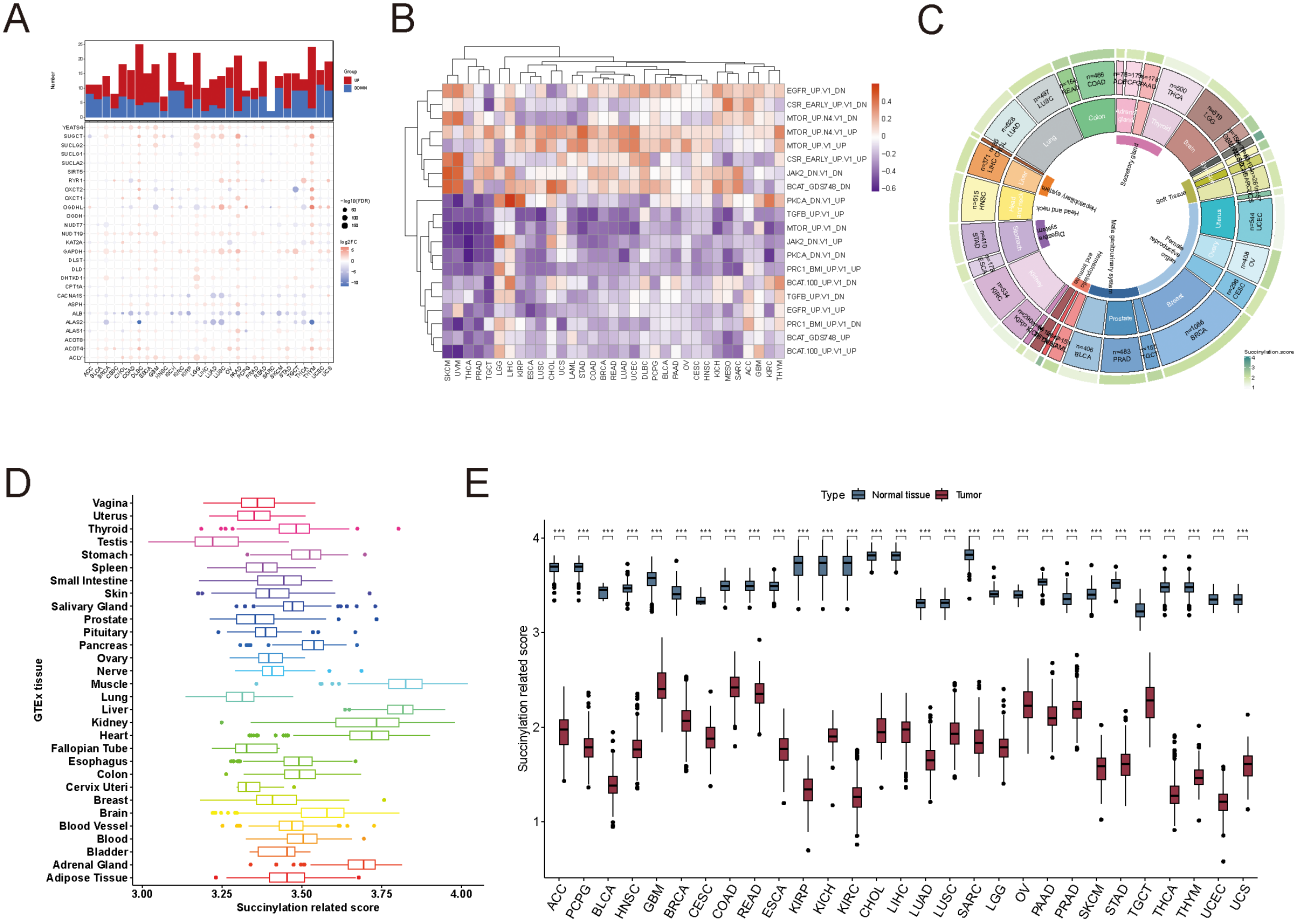
Characterization of succinylation-related molecular activities at pan-cancer level. **(A)** The expression patterns of 29 succinylation-related genes in the TCGA dataset. **(B)** Correlation analysis between succinylation scores and oncogenic signaling pathways. **(C)** Distribution of succinylation-related molecular activities. **(D)** Comparison of succinylation scores between tumor tissues in TCGA and normal tissues in GTEx. **(E)** Differences in succinylation scores between tumor and normal tissues across various TCGA cancer types. ***p < 0.001.

To investigate the genomic characteristics of the 29 succinylation-related genes across pan-cancer types, a comprehensive heatmap was generated to illustrate their expression patterns using TCGA datasets (**Figure 1A**). Among thes e genes, GAPDH exhibited upregulated expression across multiple cancer types, whereas ALB was consistently downregulated. Additionally, we further explored the genomic alterations of these genes, including copy number variations (CNVs) and somatic mutations, to assess their potential impact on succinylation-related molecular activities. As shown in **Supplementary Figure S1**, succinylation-related genes exhibited significant cancer-type specificity in genomic variations at the pan-cancer level. Among them, RYR1, CACNA1S, and OGDHL were the most frequently mutated genes, while SUCLA2 and DLST appeared to be regulated by CNV alterations. These genetic variation patterns may influence succinylation metabolism and play distinct roles in different cancer types.

To systematically quantify succinylation-related molecular activities and investigate their functional implications across different cancer types, we developed succinylation scores using GSVA. To assess the reliability of the succinylation score as a representative measure of succinylation-related molecular activities, we analyzed its correlation with oncogenic signatures in TCGA. The succinylation score showed negative correlations with oncogenic pathways regulated by JAK2, EGFR, and PKCA, while it was positively associated with mTOR signaling (**Figure 1B**).

We then calculated succinylation scores in 9,938 samples spanning 33 cancer types in TCGA. The distribution of succinylation-related molecular activities across different malignancies was shown in **Figure 1C**, where COAD, DLBC, GBM, MESO, and UVM exhibited the highest succinylation-related activities. To compare these cancer-specific patterns with normal tissues, we analyzed succinylation scores in 7,788 samples from 30 tissue types in the GTEx database (**Figure 1D**). Notably, muscle and liver tissues exhibited the highest succinylation scores in normal physiological conditions.

Importantly, in almost all TCGA cancer types, succinylation scores were significantly lower in tumor tissues compared to their matched normal tissues, which suggested that higher succinylation-related molecular activities in the tumor microenvironment may suppress tumor progression (**Figure 1E**). The consistent trend observed in TCGA and GTEx datasets further suggested that tissue type plays a critical role in determining succinylation-related molecular activities.

### 3.2 Heterogeneity of succinylation score and its association with the tumor immune microenvironment

**Figure 2:**
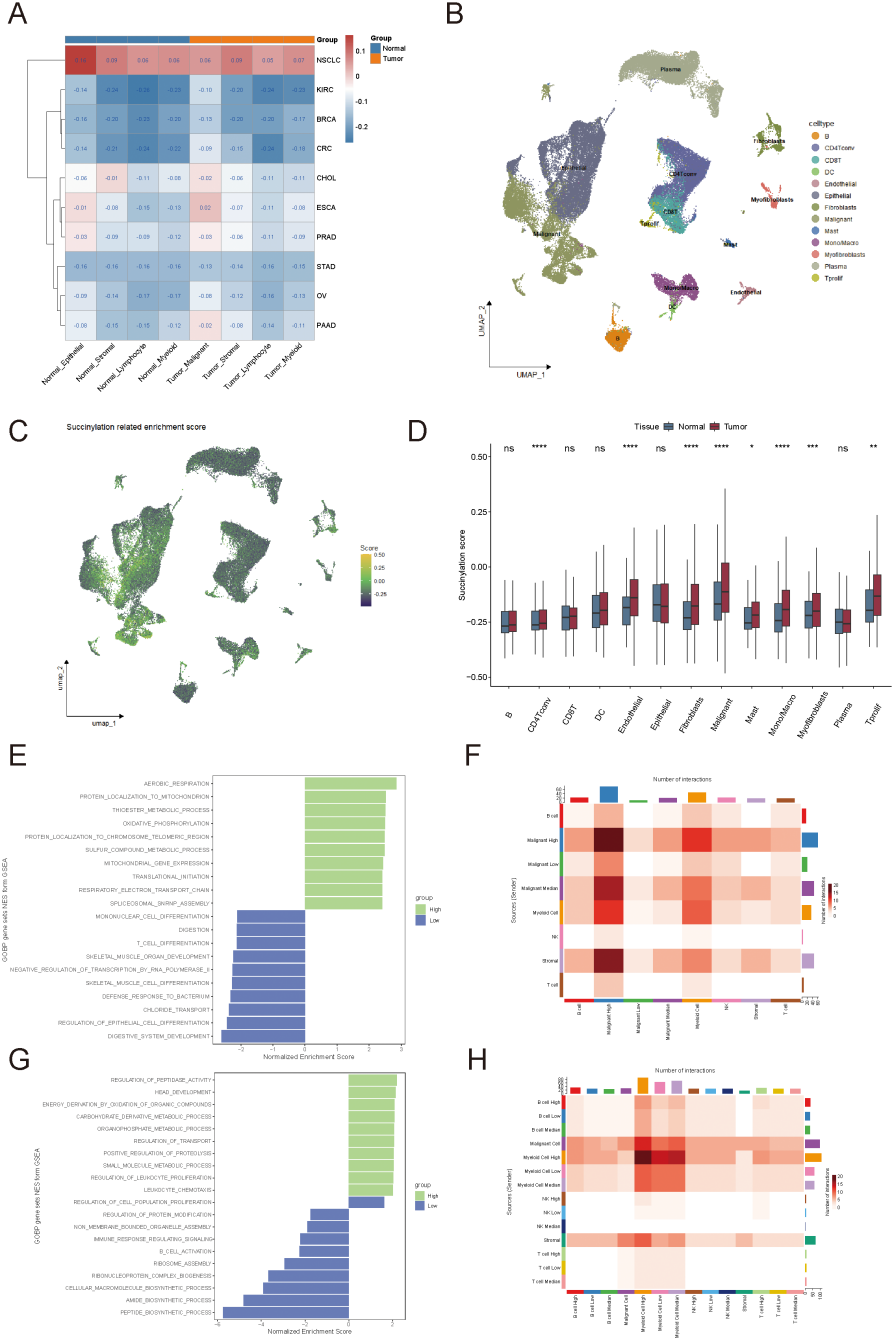
Heterogeneity of succinylation scores and their association with the tumor immune microenvironment. **(A)** Comparison of succinylation scores between tumor and normal cells across 10 solid tumor types in single-cell RNA sequencing datasets. **(B, C)** Distribution of succinylation scores among different cell types in colorectal cancer. **(D)** Succinylation scores in tumor microenvironment cell types compared to their normal counterparts. **(E)** Functional enrichment analysis of succinylation scores in tumor cells. **(F)** Cell-cell interaction analysis of tumor cells. **(G)** Functional enrichment analysis of succinylation scores in immune cells. **(H)** Cell-cell interaction analysis of immune cells. ns, not significant, *p* ≥ 0.05; *p < 0.05; **p < 0.01; ***p < 0.001.

To evaluate the heterogeneity of succinylation scores and their association with the tumor microenvironment, we utilized 10 scRNA-seq datasets, comprising 830,178 cells from 173 cancer patients across 10 solid tumor types, along with cells from 65 normal donors. CRC, ESCA, and BRCA exhibited significantly higher succinylation scores compared to their corresponding normal cells, whereas NSCLC showed lower succinylation scores in tumor cells relative to normal tissues (**Figure 2A**).

Building on the observed differences in succinylation scores among various cancer types, we focused on CRC to further explore the heterogeneity of succinylation scores by comparing distinct cell types within normal tissues and tumors (**Figures 2B, C**). As expected, most cell types in the tumor microenvironment exhibited higher succinylation scores than their counterparts in normal tissues, which suggested an enrichment of succinylation-related molecular activities in the tumor microenvironment (**Figure 2D**). This may indicate that tumor cells deplete nutrients from surrounding normal cells and consequently increase succinylation-related activity in these cells.

To investigate the impact of intratumoral heterogeneity in succinylation-related molecular activities, we analyzed succinylation scores in 66,050 single cells from 12 CRC patients (GSE166555). Tumor cells and immune cells were stratified into high-succinylation, medium-succinylation, and low-succinylation groups based on quartile distributions (high: >75%, medium: 25%–75%, low: <25%). In tumor cells, mitochondrial oxidative phosphorylation and electron transport chain activity were significantly correlated with high succinylation scores (**Figure 2E**). In contrast, lower succinylation scores were associated with immune cell differentiation, which indicated a potential role of succinylation in modulating tumor immunity (**Figure 2E**). Cell-cell interaction analysis further revealed that higher succinylation scores were positively correlated with an increased number of cell-cell interactions among tumor cells (**Figure 2F**). Tumor cells in the high-succinylation group displayed the highest number of interactions, which indicated their heightened activity within the tumor microenvironment. Immune cells in the high-succinylation group were primarily involved in tumor-associated chemical metabolism, whereas immune cells in the low-succinylation group exhibited significant upregulation of immune response and chemical reaction pathways (**Figure 2G**). Additionally, we observed an increased number of interactions between immune cells in the high-succinylation group and tumor cells, which suggested that immune cells with high succinylation scores were more strongly influenced by tumor cells (**Figure 2H**).

**Figure 3:**
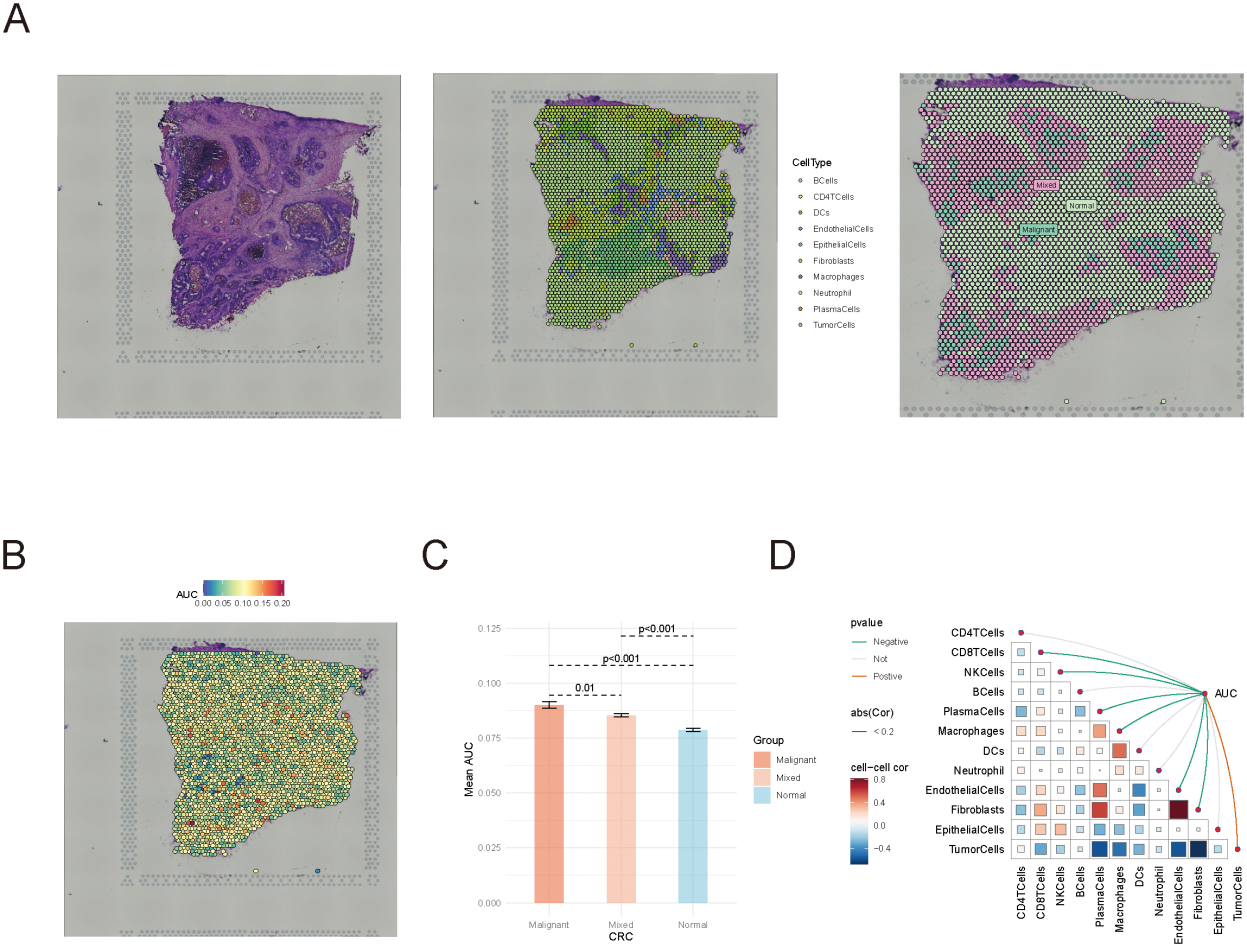
Spatial transcriptomics analysis of succinylation scores in colorectal cancer. **(A)** Classification of immune cells in spatial transcriptomics data. **(B, C)** The distribution of succinylation scores in immune cells located in tumor, mixed, and normal regions. **(D)** Spearman correlation analysis between succinylation scores and immune cell abundance.

To further examine the spatial distribution of succinylation scores within the tumor immune microenvironment, we analyzed spatial transcriptomics data from CRC patients. Immune cells were categorized based on their proximity to tumor cells, into three groups: inside malignant regions, at mixed regions, and within normal tissues (**Figure 3A**). Immune cells within the tumor region exhibited the highest average succinylation score, while those in normal tissues showed the lowest scores (**Figures 3B, C**). Notably, succinylation scores significantly increased as immune cells moved closer to tumor cells (*P* < 0.05, Wilcoxon rank sum test).

Furthermore, Spearman correlation analysis demonstrated a negative correlation between succinylation scores and immune cell abundance, whereas succinylation scores were positively correlated with tumor cell presence (**Figure 3D**). These findings underscored the potential role of succinylation-related molecular activities in shaping the tumor immune microenvironment, and highlighted the distinct metabolic status of immune cells in tumors compared to normal tissues.

### 3.3 Association between succinylation and immune checkpoints and immunomodulation in pan-cancer

**Figure 4:**
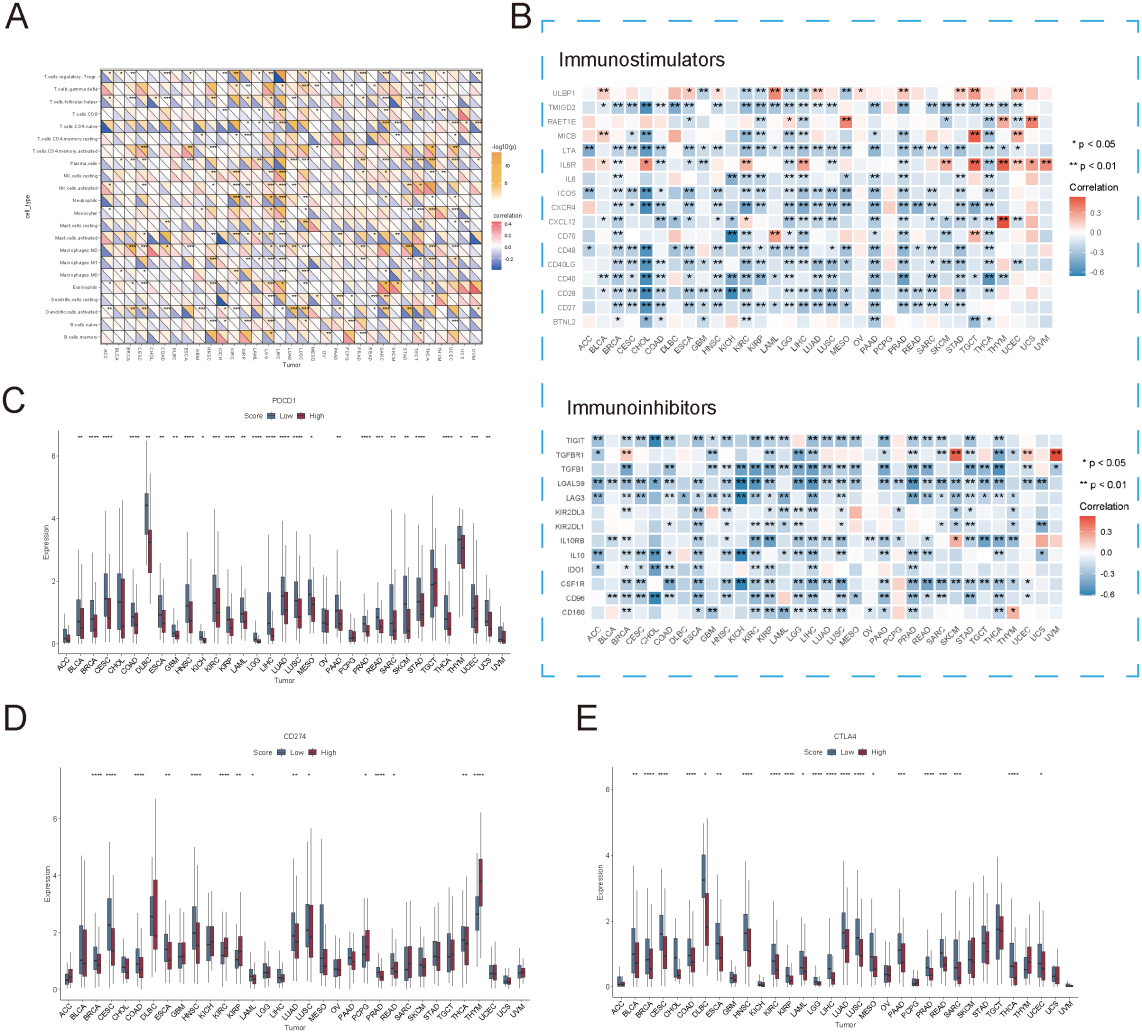
Association between succinylation scores and immune checkpoint expression. **(A)** Correlation between succinylation scores and immune cell infiltration in different cancer types. **(B)** Correlation between succinylation scores and the expression of immune-related genes (immunostimulators and immunoinhibitors) across multiple cancers. **(C–E)** Differences in PDCD1 (PD-1), CD274 (PD-L1), and CTLA4 expression between high-succinylation and low-succinylation groups. ns, not significant, *p* ≥ 0.05; *p < 0.05; **p < 0.01; ***p < 0.001; ****p < 0.0001.

To further explore the potential relationship between succinylation-related molecular activities and immune checkpoints as well as immunomodulation across various cancer types, we analyzed the correlation between succinylation scores and immune cell infiltration. As expected, immune cell abundance was significantly associated with succinylation scores in most cancer types (**Figure 4A**). However, the infiltration patterns of specific immune cell populations varied across cancer types, which lead to divergent correlations between succinylation levels and immune infiltration. For instance, in cancers such as STAD, COAD, and BRCA, higher succinylation scores were positively correlated with the infiltration of immunosuppressive M2 macrophages, which are known to promote tumor progression. However, this trend was not observed uniformly across all malignancies, which suggested that succinylation-related molecular activities may contribute to an immunosuppressive tumor microenvironment in a cancer-type-dependent manner.

To further elucidate the association between succinylation and immune modulation, we examined the correlation between succinylation scores and immune-related gene expression. As illustrated in **Figure 4B**, succinylation scores demonstrated a significant negative correlation with the expression of immune-related genes across most cancers. Notably, in cancers such as COAD, BRCA, LUAD, READ, and CESC, tumors in the low-succinylation group exhibited elevated expression levels of PDCD1 (PD-1), CD274 (PD-L1), and CTLA4 at both the transcriptional and protein levels (**Figures 4C–E**). These results suggested that tumors with lower succinylation scores exhibited increased sensitivity to immune checkpoint blockade therapy, as their immune microenvironment was enriched in checkpoint molecules that were critical for immunotherapy response.

Collectively, these findings revealed that high succinylation-related molecular activities were associated with a TME characterized by immunosuppressive features, which may contribute to poorer clinical outcomes in affected patients. However, it is important to note that tumors with high succinylation scores also exhibited significant immune infiltration and distinct immune-related molecular signatures, which indicated complex immunoregulatory effects. Given these observations, succinylation scores could serve as a valuable biomarker for identifying patients more likely to benefit from immunotherapy, which offered new insights into personalized cancer treatment strategies.

### 3.4 Succinylation as a potential predictor of immunotherapy response

**Figure 5:**
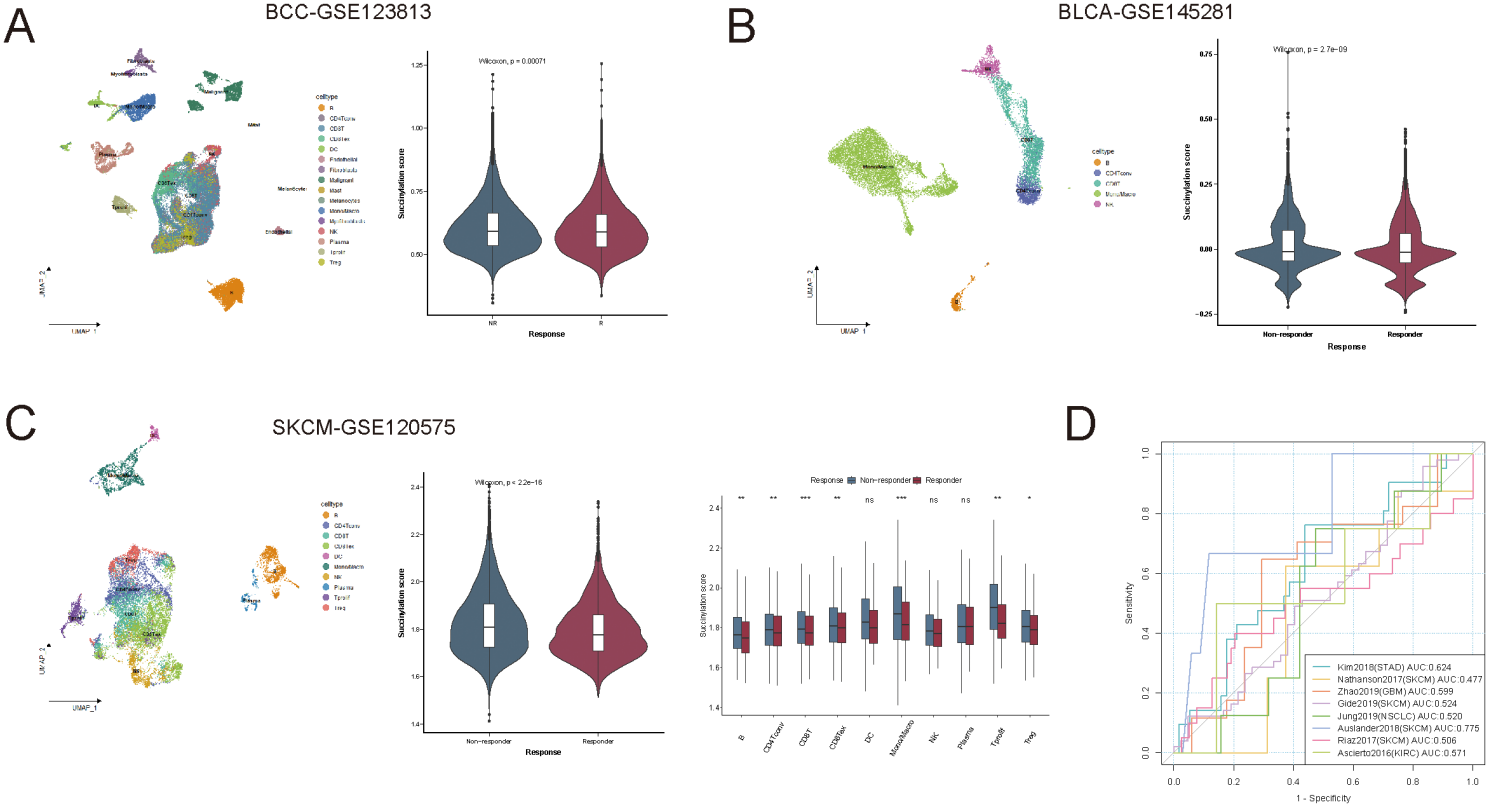
Succinylation as a predictor of immunotherapy response. **(A)** Comparison of succinylation scores between immune checkpoint blockade (ICB) responders and non-responders in a scRNA-seq dataset of basal cell carcinoma. **(B, C)** The association between succinylation scores and ICB response in blood immune cells from immunotherapy-treated patients with bladder cancer and skin cutaneous melanoma. **(D)** Receiver operating characteristic (ROC) curve of succinylation scores for predicting ICB response across eight independent immunotherapy cohorts. ns, not significant, *p* ≥ 0.05; *p < 0.05; **p < 0.01; ***p < 0.001.

The immune alterations observed in different succinylation score groups suggested that succinylation scores could serve as a potential predictor for immune checkpoint blockade (ICB) therapy response. To further explore this hypothesis, we analyzed the succinylation scores of primary tumor samples (21,256 single cells from GSE123813) in a scRNA-seq dataset of BCC, comparing ICB responders and non-responders (**Figure 5A**). As expected, the succinylation scores of cells from responders were lower than those from non-responders.

To validate this finding, we extended our analysis to peripheral blood immune cells from immunotherapy-treated patients in BLCA-GSE145281 and SKCM-GSE120575, rather than tumor-infiltrating cells. Similar to the observations in solid tumors, immune cells from ICB responders exhibited lower succinylation scores than those from non-responders (**Figures 5B, C**), which suggested that succinylation-related molecular activities also influenced systemic immune responses to immunotherapy.

We further assessed the predictive capability of succinylation scores in large RNA-seq cohorts, including 411 patients across 8 independent ICB-treated cohorts. The succinylation score achieved a mean area under the curve (AUC) of approximately 0.575 for predicting ICB response across different cancer types (**Figure 5D**), which suggested its potential as a predictive biomarker for identifying ICB responders.

Beyond immunotherapy, succinylation also influenced the efficacy of chemotherapy and targeted therapy. Using the R package “pRRophetic”, we estimated drug sensitivity values (IC50) for 237 chemotherapeutic and targeted agents from the Cancer Genome Project (CGP2016) across all TCGA samples. Despite variations among different drugs, succinylation scores exhibited significant correlations with IC50 values for chemotherapy and targeted therapies at the pan-cancer level (**Supplementary Figure S2**). Given that the effects of chemotherapy and targeted therapies were partially mediated by metabolic rewiring—which is influenced by succinylation—the impact of succinylation varies across different drugs.

Taken together, our findings suggested that succinylation scores could provide valuable insights into immunotherapy response prediction and also be relevant in the context of chemotherapy and targeted therapy. A deeper understanding of the metabolic role of succinylation in tumor progression and treatment response could further optimize therapeutic strategies across various cancer types.

### 3.5 Identification of molecular subtypes based on succinylation-related genes

**Figure 6:**
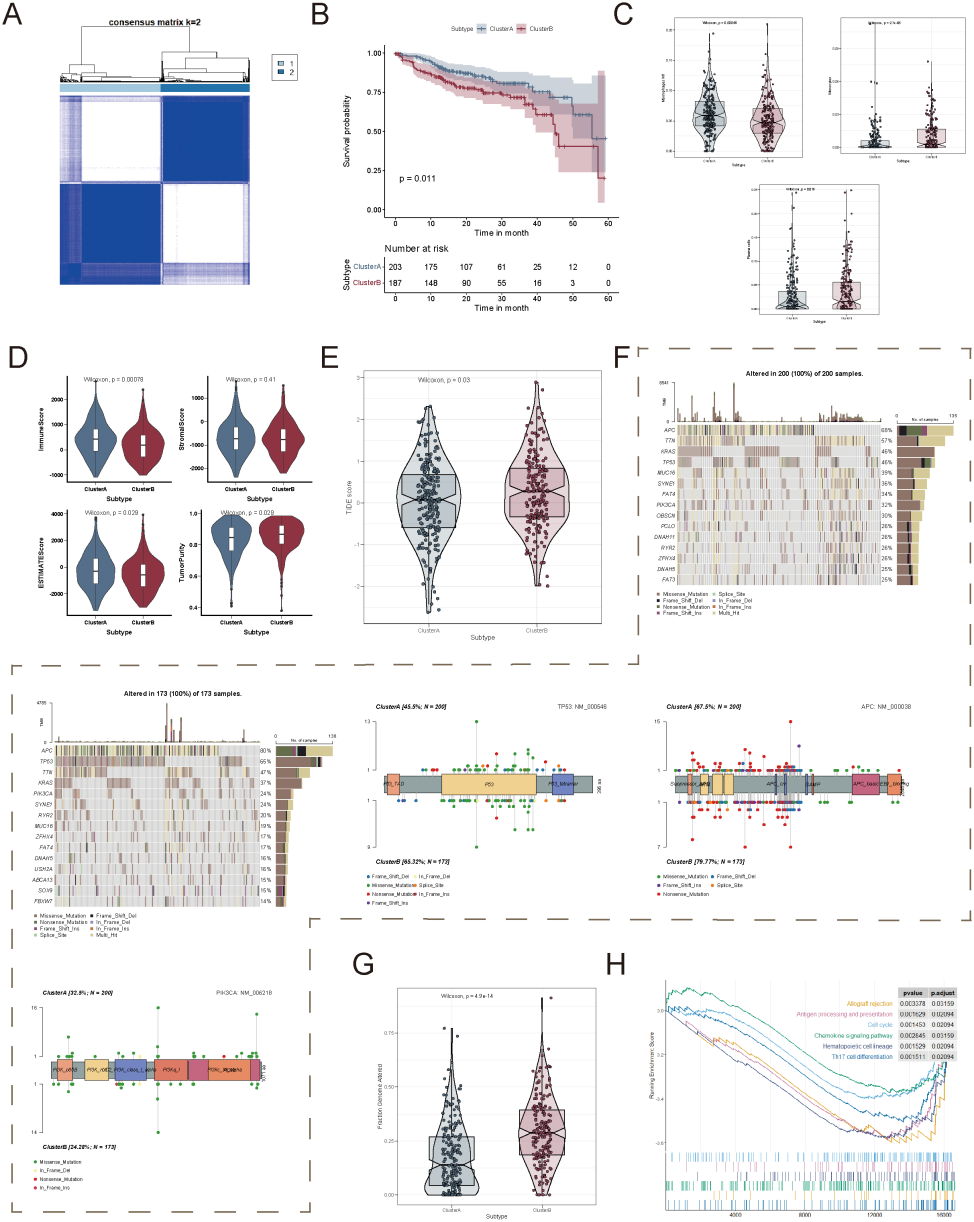
Identification of succinylation-related molecular subtypes in colorectal cancer. **(A)** Consensus clustering analysis based on succinylation-related genes. **(B)** Kaplan-Meier survival analysis between patients in Cluster A and Cluster **(B, C)** Differences in immune cell infiltration between the two succinylation-related clusters. **(D)** Comparison of four tumor microenvironment scores between the two clusters. **(E)** Comparison of Tumor Immune Dysfunction and Exclusion (TIDE) scores between the two clusters. **(F)** Mutation frequency of the genes in each succinylation subtype, along with the mutation characteristics of TP53, APC, and PIK3CA. **(G)** Comparison of the fraction of genome alterations (FGA) between the two clusters. **(H)** Gene Set Enrichment Analysis (GSEA) based on differentially expressed genes between the two clusters.

Given that CRC exhibited distinct succinylation-related metabolic and immune characteristics compared to other cancer types, we focused on classifying CRC based on succinylation-related gene expression. We conducted unsupervised clustering analysis using 29 succinylation-related genes across 430 tumor samples. The clustering matrix (k) was increased from 2 to 10 to determine the optimal number of clusters. As a result, consensus clustering indicated that k = 2 was the most suitable solution, leading to the identification of two distinct molecular subtypes, designated as Cluster A and Cluster B (**Figure 6A**). Among them, Cluster A included 229 cases, while Cluster B comprised 201 cases. Notably, survival analysis revealed a significant prognostic difference between the two subtypes, with Cluster A demonstrating a clear survival advantage (*p* = 0.01, **Figure 6B**).

To further investigate the biological significance of these molecular subtypes, we assessed immune infiltration patterns, stromal composition, and TME characteristics between Cluster A and Cluster B. Macrophages M1 infiltration was significantly elevated in Cluster A, whereas Monocytes and Plasma cells were significantly enriched in Cluster B (**Figure 6C**). In addition, immune scores were significantly higher in Cluster A, whereas tumor purity was significantly higher in Cluster B (**Figure 6D**). Given that TIDE (Tumor Immune Dysfunction and Exclusion) scores positively correlate with immune evasion and resistance to immunotherapy, we further evaluated the TIDE scores of the two clusters in the TCGA-COAD cohort. The results demonstrated that Cluster B had significantly higher TIDE scores than Cluster A, which indicated a stronger immune evasion phenotype and a lower likelihood of response to immunotherapy (**Figure 6E**).

We then examined the association between succinylation-related molecular subtypes and genomic alterations, including CNV and somatic mutations. The top 20 most frequently mutated genes were identified in both clusters (**Figure 6F**). Interestingly, Cluster A exhibited a higher mutation frequency in oncogenes, such as PIK3CA, whereas Cluster B displayed a higher mutation frequency in tumor suppressor genes, including TP53 and APC (**Figure 6F**). In terms of chromosomal alterations, Cluster B exhibited a significantly higher fraction of genome alterations (FGA), which indicated greater genomic instability (**Figure 6G**).

To gain deeper insights into the functional differences between the two succinylation subtypes, we performed Gene Set Enrichment Analysis. The results demonstrated that Cluster A was primarily enriched in immune-related pathways, which included allograft rejection, antigen processing and presentation, chemokine signaling pathway, and Th17 cell differentiation (**Figure 6H**). These findings highlighted that succinylation-related molecular subtypes were strongly associated with tumor immune modulation and genomic heterogeneity, and they potentially influenced tumor progression and therapeutic responses.

### 3.6 Construction and validation of the succinylation-related prognostic model in CRC

To evaluate the prognostic significance of succinylation-related molecular activities, we first examined the association between succinylation scores and overall survival across multiple cancer types. A significant difference in OS was observed between the low-succinylation and high-succinylation groups (stratified by the median succinylation score) at the pan-cancer level (**Supplementary Figure S3**). Further survival analysis confirmed that eight specific cancer types exhibited statistically significant OS differences between these two groups (**Supplementary Figure S3**).

**Figure 7:**
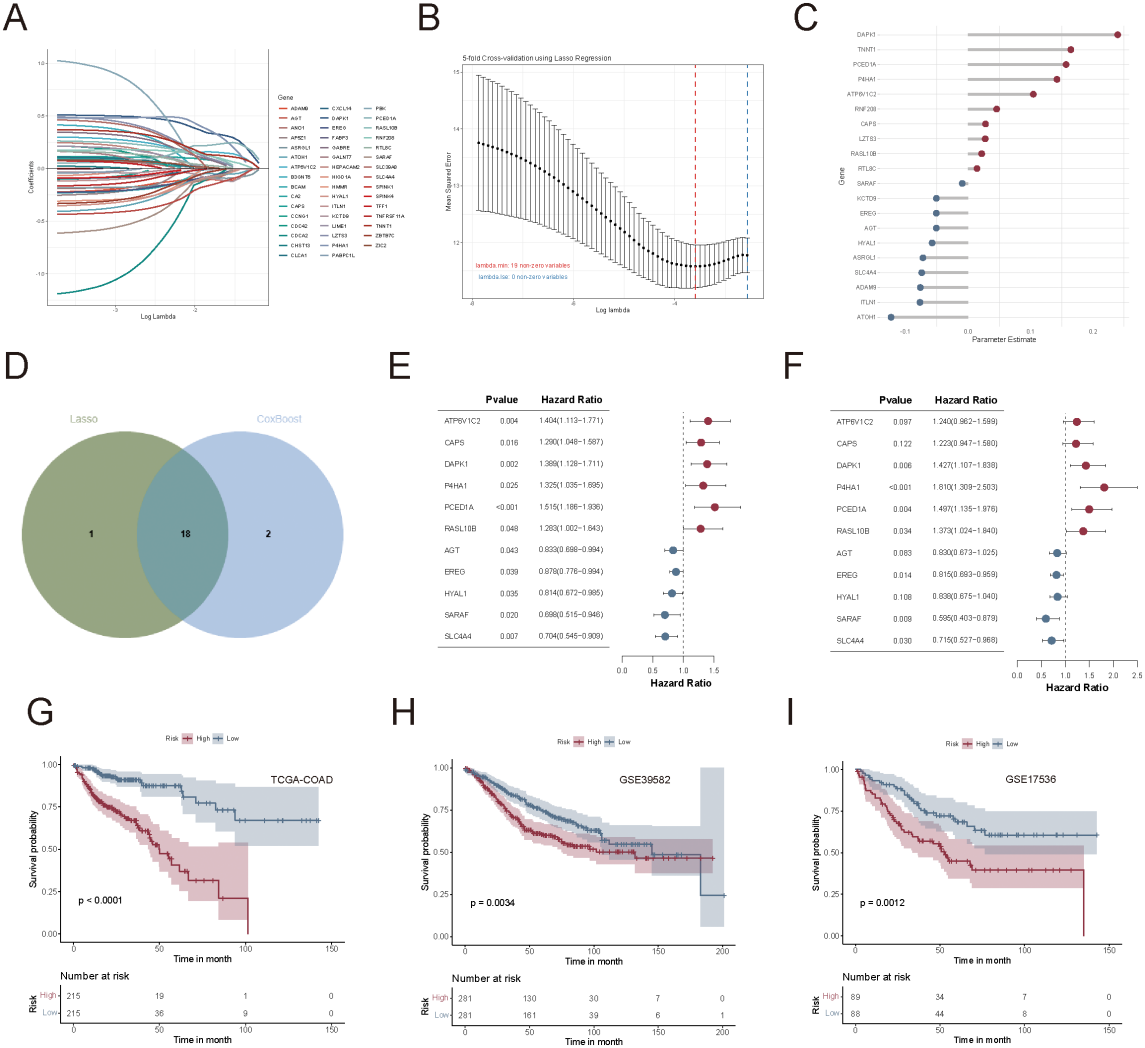
Construction and validation of the succinylation-related prognostic model in colorectal cancer. **(A)** Univariate Cox regression identified 48 prognostically relevant genes from 596 subtype-related genes. **(B)** Lasso-Cox regression analysis for feature selection. **(C)** CoxBoost regression analysis for feature selection. **(D)** Venn diagram of two sets of selected genes. **(E, F)** Univariate and multivariate forest plots of the 11-gene prognostic model. **(G–I)** Kaplan-Meier survival curves between high-risk and low-risk groups in TCGA-COAD, GSE39582, and GSE17536 cohorts.

To develop a simplified succinylation-based prognostic model and identify key genes associated with prognosis, we used the TCGA-COAD cohort (n = 430) and applied a Cox proportional hazards regression model combined with three machine-learning algorithms. Among 596 subtype-related genes, 48 genes were initially identified as prognostically relevant via univariate Cox regression analysis (**Figure 7A**). Lasso-Cox regression further refined the selection to 19 genes (**Figure 7B**), while the CoxBoost algorithm identified 20 genes (**Figure 7C**). A Venn diagram intersection analysis revealed 18 core biomarkers (**Figure 7D**), from which a final set of 11 genes (ATP6V1C2, CAPS, DAPK1, P4HA1, PCED1A, RASL10B, AGT, EREG, HYAL1, SARAF, and SLC4A4) was retained through stepAIC Cox regression for model construction (**Figures 7E, F**).

The succinylation-related prognostic risk score was defined as follows: succinylation predictor = expression of ATP6V1C2*(0.2153) + expression of CAPS*(0.2015) + expression of DAPK1*(0.3553) + expression of P4HA1*(0.5932) + expression of PCED1A *(0.4037) + expression of RASL10B*(0.3168) + expression of AGT*(-0.1860) + expression of EREG*(-0.2046) + expression of HYAL1*(-0.1769) + expression of SARAF*(-0.5189) + expression of SLC4A4*(-0.3361). Patients were then stratified into high-risk and low-risk groups based on the median risk score. Kaplan-Meier survival analysis demonstrated that patients in the low-risk group had significantly better OS than those in the high-risk group, as observed in the TCGA-COAD (*p* < 0.0001, **Figure 7G**), GSE39582 (*p* = 0.0034, **Figure 7H**), and GSE17536 (*p* = 0.0012, **Figure 7I**) cohorts.

**Figure 8:**
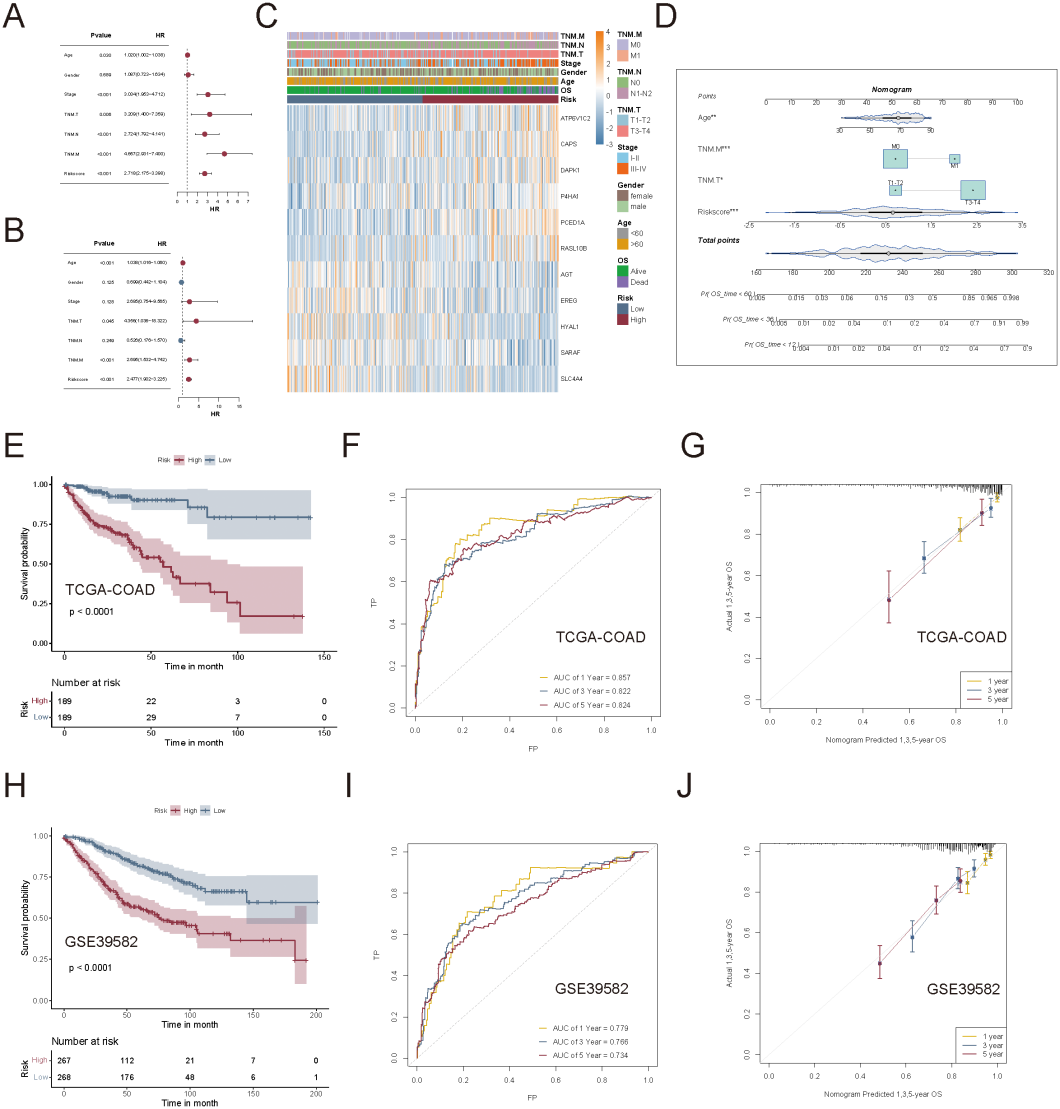
Evaluation of the clinical applicability of the succinylation-based prognostic model. **(A, B)** Univariate and multivariate Cox regression analyses to compare the prognostic value of the succinylation-based risk score and common clinical characteristics. **(C)** Heatmap to display the relationship between clinical characteristics, risk score distribution, and key gene expression patterns. **(D)** Nomogram of the succinylation-based prognostic model incorporating risk score and clinical parameters (Age, TNM.T, and TNM.M). **(E)** Kaplan-Meier analysis curves between high-risk and low-risk groups. **(F)** Time-dependent ROC curves to illustrate the predictive accuracy of the nomogram-based model. **(G)** Calibration curves to confirm the robustness of the nomogram. **(H–J)** External validation of the prognostic nomogram in the GSE39582 cohort.

Univariate and multivariate Cox regression analyses confirmed that risk scores were independent prognostic factors for CRC, which outperformed common clinical variables such as age, TNM staging, and metastasis status (**Figures 8A, B**). A heatmap summarizing the clinical characteristics, risk score distribution, and key gene expression profiles in COAD patients from TCGA was presented in **Figure 8C**.

To assess the clinical applicability of the succinylation risk model, we integrated age, TNM.T, and TNM.M staging into a nomogram for predicting 1-, 3-, and 5-year OS in CRC patients using the TCGA-COAD dataset (**Figure 8D**). The nomogram-based model demonstrated superior prognostic performance compared to the gene signature alone. Kaplan-Meier analysis revealed a significant survival difference between the high- and low-risk groups (*p* < 0.0001, **Figure 8E**). The time-dependent AUC for 1-, 3-, and 5-year survival was 0.857, 0.822, and 0.824, respectively (**Figure 8F**), which showed high predictive accuracy. Calibration curves confirmed the robustness and reliability of the model in predicting long-term survival outcomes (**Figure 8G**). Furthermore, the nomogram was independently validated in the GSE39582 cohort, demonstrating high accuracy in predicting OS across different time points (**Figures 8H-J**).

Taken together, these findings underscored that succinylation-related molecular activities were strongly associated with CRC prognosis. Moreover, the succinylation-based prognostic model exhibited significant clinical relevance across multiple independent cohorts, which indicated that ATP6V1C2, CAPS, DAPK1, P4HA1, PCED1A, RASL10B, AGT, EREG, HYAL1, SARAF, and SLC4A4 hold potential as robust prognostic biomarkers in CRC.

### 3.7 Preliminary exploration of PCED1A function in CRC

**Figure 9:**
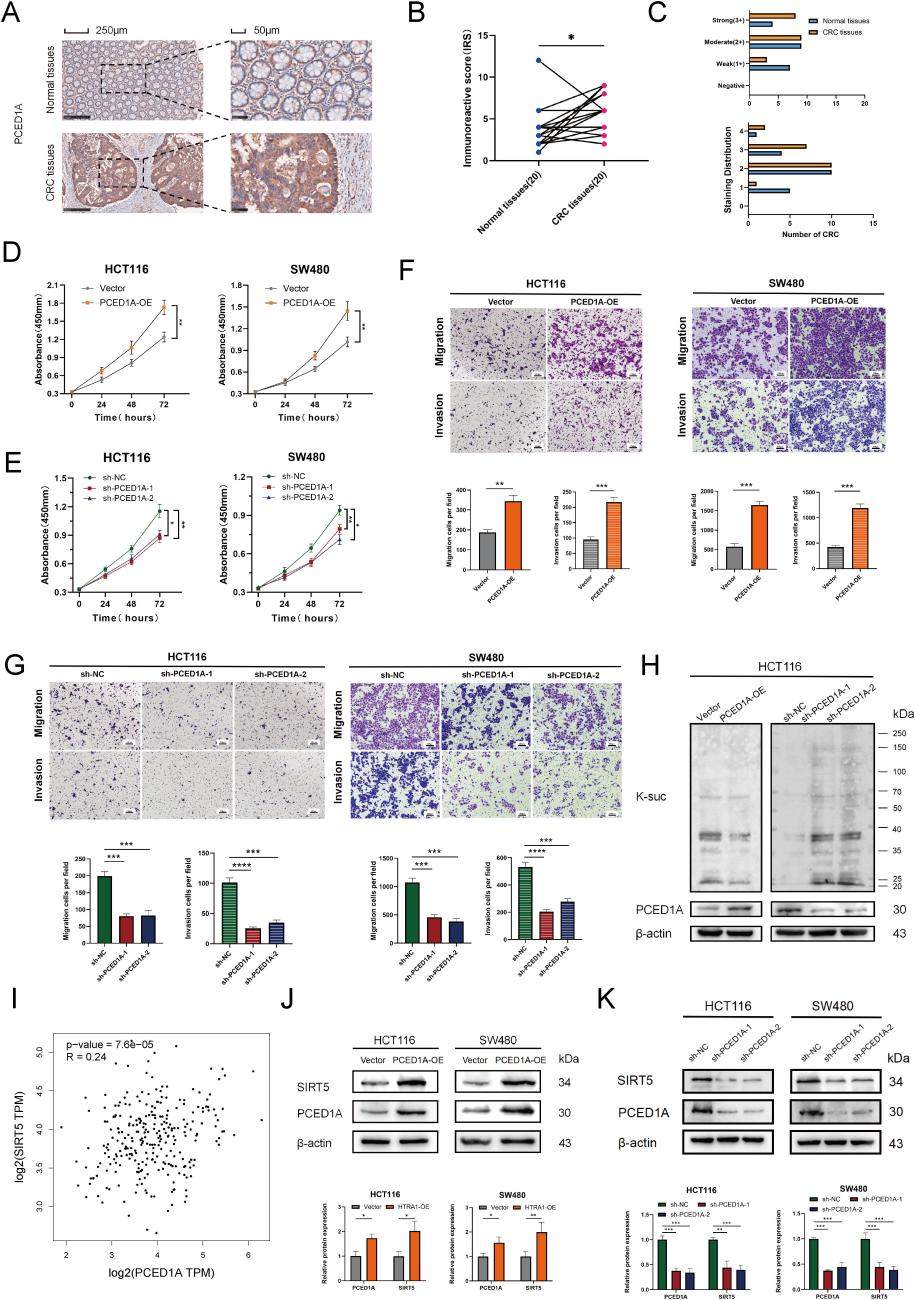
Functional validation of PCED1A in CRC. **(A)** Representative immunohistochemistry (IHC) images. **(B)** Immunoreactive Scores (IRS) of 20 paired paraffin-embedded tissue sections. **(C)** Staining intensity score (0–3) and staining distribution score (0-4). **(D, E)** Cell proliferation curve of SW480 and HCT116 cells. **(F, G)** Representative Transwell migration and invasion assay images of SW480 and HCT116 cells, with quantification of migrated and invaded cells. **(H)** Effect of PCED1A expression levels on succinyllysine modification. **(I)** Correlation analysis between PCED1A and SIRT5 expression from the GEPIA2 online database (http://gepia2.cancer-pku.cn/). **(J, K)** Western blotting to detect the protein levels of PCED1A and SIRT5, with quantification of relative protein expression. *p < 0.05; **p < 0.01; ***p < 0.001; ****p<0.0001.

Using the TCGA-COAD dataset, we analyzed the differential expression of the 11 genes in the succinylation-based prognostic model between tumor and adjacent normal tissues, as well as their individual prognostic significance. The results showed that all genes exhibited significant differential expression in tumor tissues; however, only four genes (CAPS, DAPK1, P4HA1, and PCED1A) demonstrated independent prognostic value (**Supplementary Figure S4**). Based on a literature review, we selected PCED1A, which has not been previously reported in CRC, for further molecular functional investigation. We first employed IHC to assess PCED1A expression at the protein level. The results showed that, compared to adjacent normal tissues, PCED1A staining in tumor tissues had higher scores both in intensity and extent, indicating elevated protein expression in tumor tissues (**Figures 9A-C**). Subsequently, we selected two CRC cell lines with high glycolytic activity (SW480 and HCT116) (16) for PCED1A overexpression and knockdown interventions to examine its effects on CRC cell functions. As shown in **Figures 9D-G**, enhanced PCED1A expression in CRC cells led to increased proliferation, migration, and invasion capabilities, while knockdown exhibited the opposite trend, which indicated the oncogenic potential of PCED1A. Then, Western blotting revealed that PCED1A overexpression reduced overall protein succinylation, whereas PCED1A knockdown increased it (**Figure 9H**). Given that SIRT5 is considered a global regulator of lysine succinylation in mitochondria (17), we performed correlation analysis between PCED1A and SIRT5 using the TCGA-COAD mRNA dataset to explore the relationship between PCED1A and the overall succinylation levels in CRC. The results revealed a positive association between PCED1A and the desuccinylase SIRT5 in CRC tumors (**Figure 9I**). To further elucidate the mechanism linking PCED1A to succinylation, we examined SIRT5 levels in total protein extracts from PCED1A-overexpressing and knockdown CRC cells. The results showed that PCED1A influenced SIRT5 expression (**Figures 9J, K**), which provided insight into its potential regulatory role in CRC pathogenesis.

## 4 Discussion

As a crucial post-translational regulatory mechanism, succinylation garners significant attention for its role in cancer progression (18). In gastric and lung adenocarcinomas, global analyses of succinylation identify numerous succinylated proteins, providing novel insights into tumorigenesis and metabolic adaptations (5, 19). Lysine succinylation influences tumor cell functions through various pathways, including mitochondrial metabolism, gene transcription, and RNA repair (18). Additionally, succinylation acts synergistically with other post-translational modifications. Histone acetyltransferase 1 (HAT1) is recognized as a pivotal succinyltransferase during tumorigenesis, targeting a wide range of succinylated proteins distributed across subcellular compartments and participating in complex cellular processes (20). While succinylation plays a critical role in cancer progression, its regulatory functions in the tumor immune microenvironment and its prognostic implications remain largely unexplored in pan-cancer contexts.

This study systematically analyzed the molecular activities of succinylation-related genes and unveiled the critical biological significance of succinylation scores in both pan-cancer datasets and CRC. The findings demonstrated a strong association between succinylation level and the tumor microenvironment, with significant heterogeneity observed across both pan-cancer and single-cell levels. Notably, cells with high succinylation scores were closely linked to mitochondrial oxidative phosphorylation and the electron transport chain, while cells with low succinylation scores were predominantly involved in immune cell differentiation. This metabolic dichotomy reflected distinct tumor cell strategies: high succinylation activity supporting bioenergetic demands, while low succinylation activity may facilitate immune evasion by modulating inflammatory responses. Moreover, single-cell analysis revealed that tumor cells with elevated succinylation scores exhibited enhanced intercellular communication with immune cells, particularly through pathways related to metabolite transport and chemical metabolism. Given that tumor cells often engage in metabolic crosstalk with the surrounding immune cells (21), succinylation-related metabolic shifts played a role in shaping the immunosuppressive tumor microenvironment. Spatial transcriptomic data revealed that succinylation activity increased in immune cells located closer to tumor cells, suggesting localized metabolic reprogramming that may influence immune cell polarization and function.

Interestingly, while succinylation levels were elevated in tumor-proximal immune cells, overall succinylation scores in CRC showed a negative correlation with the expression of immune checkpoint molecules such as PDCD1 (PD-1), CD274 (PD-L1), and CTLA4. This apparent paradox may reflect the complex and spatially dynamic metabolic landscape of the TME. One possible explanation is metabolic competition, whereby tumor cells deplete key nutrients such as glucose and glutamine, imposing metabolic stress on nearby immune cells. This stress can impair mitochondrial function and lead to accumulation of succinyl-CoA, promoting stress-induced lysine succinylation in immune cells (18). Additionally, metabolic interference may also contribute. Excess tumor-derived succinate can be taken up by immune cells via MCT1, inhibiting succinyl-CoA synthetase and suppressing glucose oxidation, which in turn impairs T cell effector function and induces a dysfunctional phenotype (22).

Moreover, our pan-cancer analysis revealed that the negative correlation between global succinylation scores and immune checkpoint gene expression is not limited to CRC, but extends to multiple tumor types. While this pattern may seem to contrast with recent findings in melanoma—where succinylation of PD-L1 at lysine 129 was reported to promote its degradation and enhance antitumor immunity (23)—it is important to recognize that the immunological effects of succinylation are likely highly context- and cell type–dependent. A separate recent study further supports this complexity by demonstrating that elevated succinylation in immune tissues can actually impair antitumor immunity. Specifically, Hu et al. (24) showed that SIRT7, an NAD^+^-dependent lysine desuccinylase, is essential for maintaining T cell function. In T cell–specific Sirt7 knockout mice, loss of SIRT7 led to hyper-succinylation of enzymes involved in branched-chain amino acid (BCAA) catabolism, resulting in metabolic dysregulation (including acyl-CoA and fatty acid accumulation), T cell exhaustion, and reduced IFN-γ secretion. These findings underscore that elevated succinylation within T cells can suppress antitumor responses under metabolic stress. Together, these observations suggest that succinylation may exert dual and context-specific roles in modulating antitumor immunity. While site-specific succinylation events may promote immune activation in certain tumor types, elevated global succinylation, particularly in immune cells, may reflect or even contribute to an immunosuppressive phenotype. This duality likely arises from differences in dominant succinylation targets, regulatory enzymes, and metabolic states across tumor types. Future studies focusing on cell type–specific succinylation landscapes and their downstream functional consequences will be essential to fully elucidate the immunological roles of succinylation within the tumor microenvironment.

Furthermore, we constructed an 11-gene CRC prognostic model based on risk scores, with these 11 genes identified as differentially expressed between high and low CRC groups classified by succinylation scores. ATP6V1C2, a subunit of vacuolar ATPase, mediates intracellular organelle acidification via ATP hydrolysis and has been reported to promote CRC progression by activating Wnt signaling and driving EMT (25). CAPS and SARAF are both calcium signaling regulators. CAPS modulates vesicle exocytosis in a calcium-dependent manner (26), while SARAF fine-tunes store-operated calcium entry (SOCE) and downstream transcriptional responses (27). Calcium-mediated remodeling of mitochondrial metabolism, as recently described (28), may in turn influence lysine succinylation by altering redox state and tricarboxylic acid cycle dynamics. DAPK1, a serine/threonine kinase, participates in oxidative stress regulation (29). Oxidative stress has been shown to reshape mitochondrial metabolism and promote the accumulation of intermediates such as succinyl-CoA (30), suggesting that succinylation may serve as a post-translational marker of metabolic stress. P4HA1, an enzyme involved in collagen hydroxylation and hypoxia response, has been recently shown to regulate succinylation in glioblastoma by increasing intracellular succinate and enhancing PGK1 K191/K192 succinylation, which stabilizes PGK1 and promotes aerobic glycolysis via the HIF1α/ATF3 axis (31). RASL10B, a RAS-like GTPase family member, has been identified as a right-sided CRC-specific prognostic marker. High expression is significantly associated with poor survival, suggesting a role in tumor progression and subtype-specific stratification (32). AGT and SLC4A4 are implicated in oxidative and metabolic stress. AGT uptake induces reactive oxygen species (ROS) production in epithelial cells independently of the classical angiotensin signaling pathway (33). SLC4A4, a Na^+^/HCO_3_^-^ cotransporter, when dysregulated, leads to intracellular alkalinization and impaired mitochondrial function in β cells (34). These metabolic disturbances may promote succinylation by increasing succinyl-CoA levels or triggering stress-induced non-enzymatic modifications. EREG, an EGFR ligand upregulated in CRC and intestinal stem cell niches, contributes to tumor progression and metabolic adaptation (35, 36). Through EGFR-mediated signaling and microenvironmental remodeling, EREG may influence mitochondrial metabolism and succinate accumulation, thereby potentially affecting succinylation dynamics under oncogenic or regenerative conditions. HYAL1, a lysosomal hyaluronidase involved in hyaluronic acid degradation, has been shown to suppress CRC metastasis by modulating the MMPs/TIMPs balance and F-actin organization, thereby inhibiting cell invasion and migration (37).

Although research on PCED1A remains limited, emerging evidence suggests its involvement in fatty acid metabolism and obesity development (38). Obesity often accompanies metabolic disorders and mitochondrial dysfunction, and succinylation, as a key regulator of mitochondrial metabolism, may play a critical role. In our study, functional assays revealed that PCED1A overexpression significantly promoted CRC cell proliferation, migration, and invasion, while its knockdown exerted the opposite effects. These phenotypes are not only hallmarks of cancer progression but are also known to be regulated by succinylation, particularly considering its intimate relationship with energy metabolism (8, 39). In line with this, we observed that PCED1A overexpression reduced global protein succinylation levels, whereas PCED1A knockdown increased them, further supporting a role for PCED1A in modulating cellular succinylation dynamics. PCED1A encodes a protein containing a PC-esterase domain, suggesting potential enzymatic functions related to esterification, acylation, or deacylation (40). Notably, PCED1A upregulation was associated with increased SIRT5 expression (**Figures 9J, K**), raising the possibility that PCED1A may enhance desuccinylation at least partially through promoting SIRT5 expression. Previous studies have established SIRT5 as a master regulator of lysine succinylation across mitochondrial metabolic pathways, with SIRT5 deficiency leading to widespread hyper-succinylation and metabolic imbalance (17). Considering the broader role of SIRT5 in regulating oxidative stress responses and mitochondrial adaptation, future investigations are warranted to elucidate the molecular mechanisms underlying PCED1A-mediated SIRT5 upregulation and their contribution to mitochondrial plasticity and tumor metabolic reprogramming in CRC (41).

Although this study systematically analyzed the significance of succinylation scores in pan-cancer and CRC, several limitations should be acknowledged. First, the study primarily relies on bioinformatics analyses of publicly available databases. While the prognostic model demonstrated stability in the validation cohort, further experimental validation in larger clinical sample sets—with detailed clinical records—is necessary to minimize potential confounding factors. Second, the underlying mechanisms of succinylation scores in the tumor microenvironment, particularly their specific associations with immune evasion and metabolic reprogramming, require further investigation. Lastly, although we experimentally demonstrated that PCED1A overexpression promotes global protein desuccinylation and upregulates SIRT5 levels in CRC cells, the precise regulatory mechanisms—such as whether PCED1A directly modulates SIRT5 transcription, stability, or enzymatic activity—are still unclear. Future studies should focus on dissecting the molecular pathways by which PCED1A influences the SIRT5–succinylation axis and determining its broader impact on mitochondrial function and tumor metabolic adaptation.

## 5 Conclusions

Succinylation introduces a new perspective on metabolic regulation in tumor progression. Not only broader biological implications but also novel therapeutic strategies targeting succinylation warrant further exploration. In this study, we provided a comprehensive pan-cancer analysis of succinylation-related molecular activities and showed that distinct succinylation patterns correlate with the survival outcomes of patients with various cancers. According to immune infiltration, single-cell, and spatial transcriptomic analyses, succinylation may also participate in shaping the tumor immune microenvironment, nevertheless, by an unidentified mechanism. CRC displayed a unique succinylation-related molecular signature, and PCED1A was identified as a key gene. PCED1A upregulated SIRT5 expression, which suggested a potential role in desuccinylation-mediated metabolic regulation. The underlying connection between PCED1A and succinylation in CRC requires further investigation. Collectively, the current findings begin to help us understand the crucial role of succinylation in tumor biology and provide a novel direction for succinylation-based therapeutic strategies in cancer.

## Data Availability

The original contributions presented in the study are included in the article/**Supplementary Material**. Further inquiries can be directed to the corresponding author.

## Supplementary material

Supplementary Figure 1 Genomic alterations of succinylation-related genes at pan-cancer level. **(A)** The distribution of somatic mutations in succinylation-related genes. **(B)** Somatic mutation frequency of succinylation-related genes. **(C)** Correlation between Copy number variation (CNV) alterations and gene expression levels. **(D)** CNV landscape of 29 succinylation-related genes.

Supplementary Figure 2 Correlation analysis between succinylation scores and drug sensitivity at the pan-cancer level. **(A)** Correlation between Genomics of Drug Sensitivity in Cancer (GDSC) drug sensitivity and mRNA expression of succinylation-related genes. **(B)** Correlation between Cancer Therapeutics Response Portal (CTRP) drug sensitivity and mRNA expression of succinylation-related genes.

Supplementary Figure 3 Survival analysis of succinylation scores in different cancer types. **(A)** Kaplan-Meier overall survival (OS) curves for adrenocortical carcinoma. **(B)** Kaplan-Meier OS curves for breast invasive carcinoma. **(C)** Kaplan-Meier OS curves for colon adenocarcinoma. **(D)** Kaplan-Meier OS curves for low-grade glioma. **(E)** Kaplan-Meier OS curves for liver hepatocellular carcinoma. **(F)** Kaplan-Meier OS curves for mesothelioma. **(G)** Kaplan-Meier OS curves for pancreatic adenocarcinoma. **(H)** Kaplan-Meier OS curves for testicular germ cell tumors.

Supplementary Figure 4 Differential expression and prognostic significance of succinylation-related prognostic genes in CRC. **(A)** Differential expression analysis of 11 succinylation-related prognostic genes. **(B)** Kaplan-Meier overall survival (OS) curves for CAPS in CRC patients. **(C)** Kaplan-Meier OS curves for DAPK1 in CRC patients. **(D)** Kaplan-Meier OS curves for P4HA1 in CRC patients. **(E)** Kaplan-Meier OS curves for PCED1A in CRC patients. ***p* < 0.01; ****p* < 0.001;****p* < 0.0001.
