# Supplementary figures and images for: Succinylation-related molecular activities in cancer: metabolic adaptations, immune landscape, and prognostic significance in colorectal cancer
Source: Front Immunol. 2025 May 20;16:1571446. doi: 10.3389/fimmu.2025.1571446 (PMC12129993; doi:10.3389/fimmu.2025.1571446)

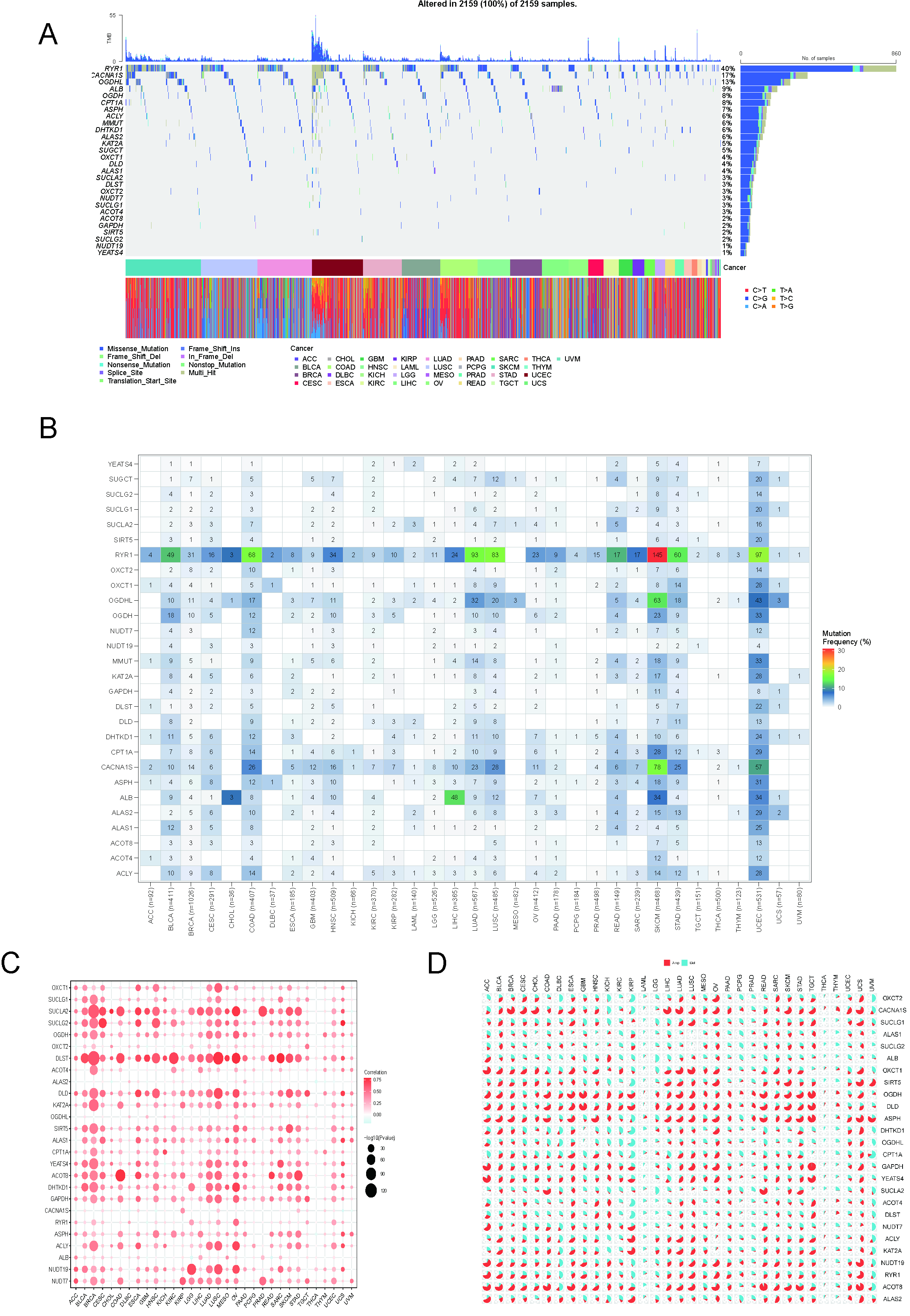

Supplement: Supplementary Figure 1 — Genomic alterations of succinylation-related genes at pan-cancer level. (A) The distribution of somatic mutations in succinylation-related genes. (B) Somatic mutation frequency of succinylation-related genes. (C) Correlation between Copy number variation (CNV) alterations and gene expression levels. (D) CNV landscape of 29 succinylation-related genes. [file Image1.tif]

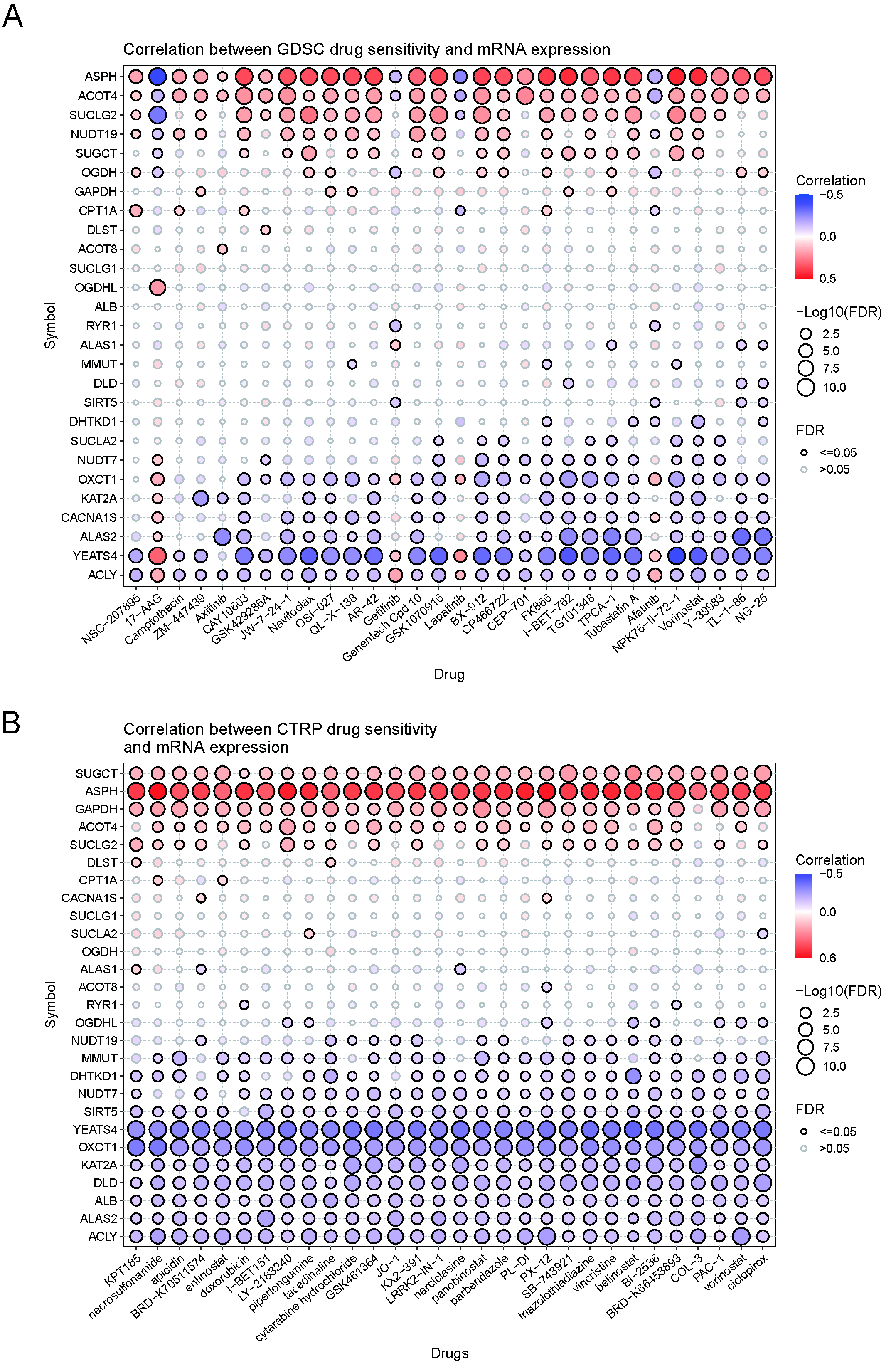

Supplement: Supplementary Figure 2 — Correlation analysis between succinylation scores and drug sensitivity at the pan-cancer level. (A) Correlation between Genomics of Drug Sensitivity in Cancer (GDSC) drug sensitivity and mRNA expression of succinylation-related genes. (B) Correlation between Cancer Therapeutics Response Portal (CTRP) drug sensitivity and mRNA expression of succinylation-related genes. [file Image2.tif]

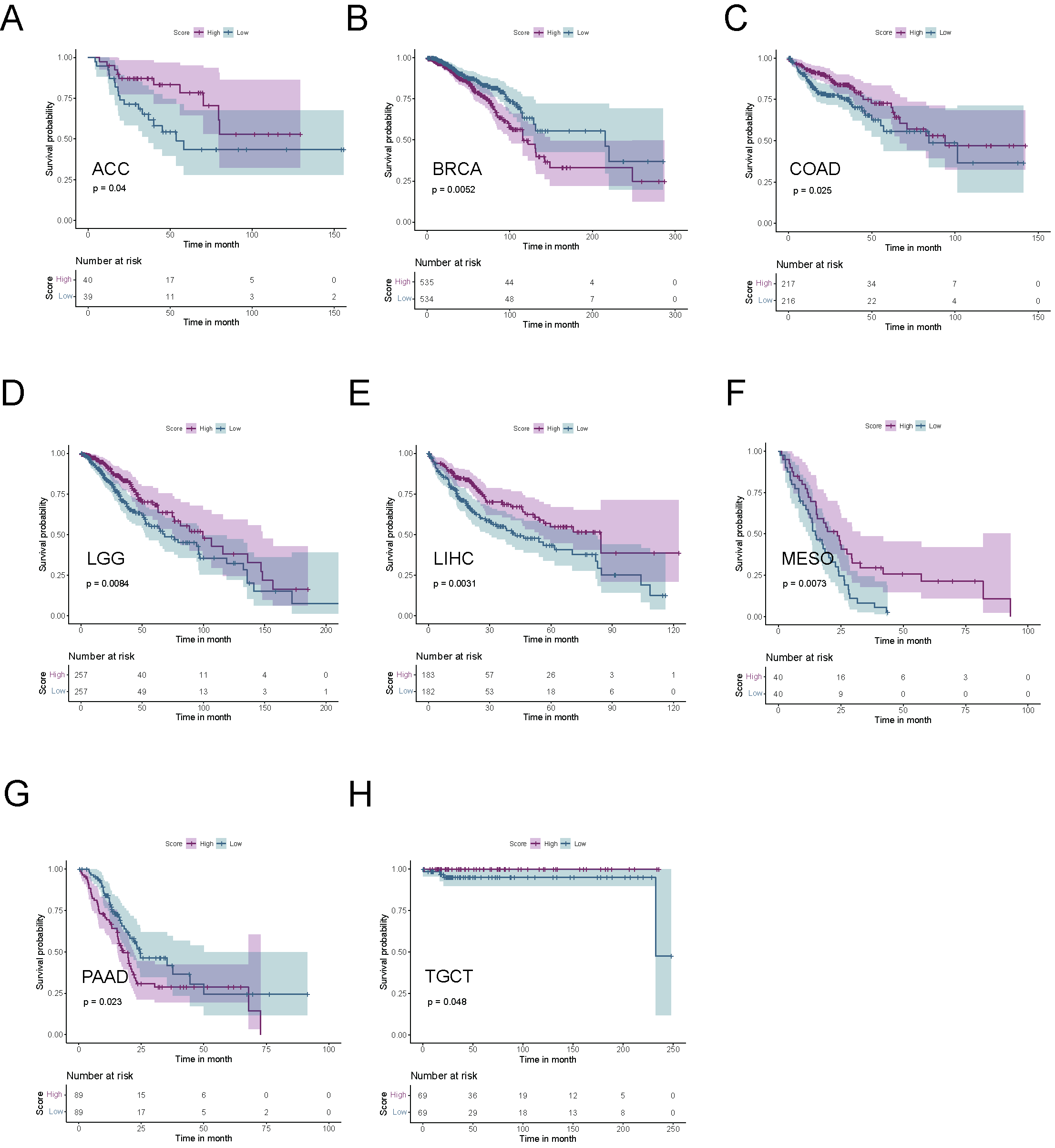

Supplement: Supplementary Figure 3 — Survival analysis of succinylation scores in different cancer types. (A) Kaplan-Meier overall survival (OS) curves for adrenocortical carcinoma. (B) Kaplan-Meier OS curves for breast invasive carcinoma. (C) Kaplan-Meier OS curves for colon adenocarcinoma. (D) Kaplan-Meier OS curves for low-grade glioma. (E) Kaplan-Meier OS curves for liver hepatocellular carcinoma. (F) Kaplan-Meier OS curves for mesothelioma. (G) Kaplan-Meier OS curves for pancreatic adenocarcinoma. (H) Kaplan-Meier OS curves for testicular germ cell tumors. [file Image3.tif]

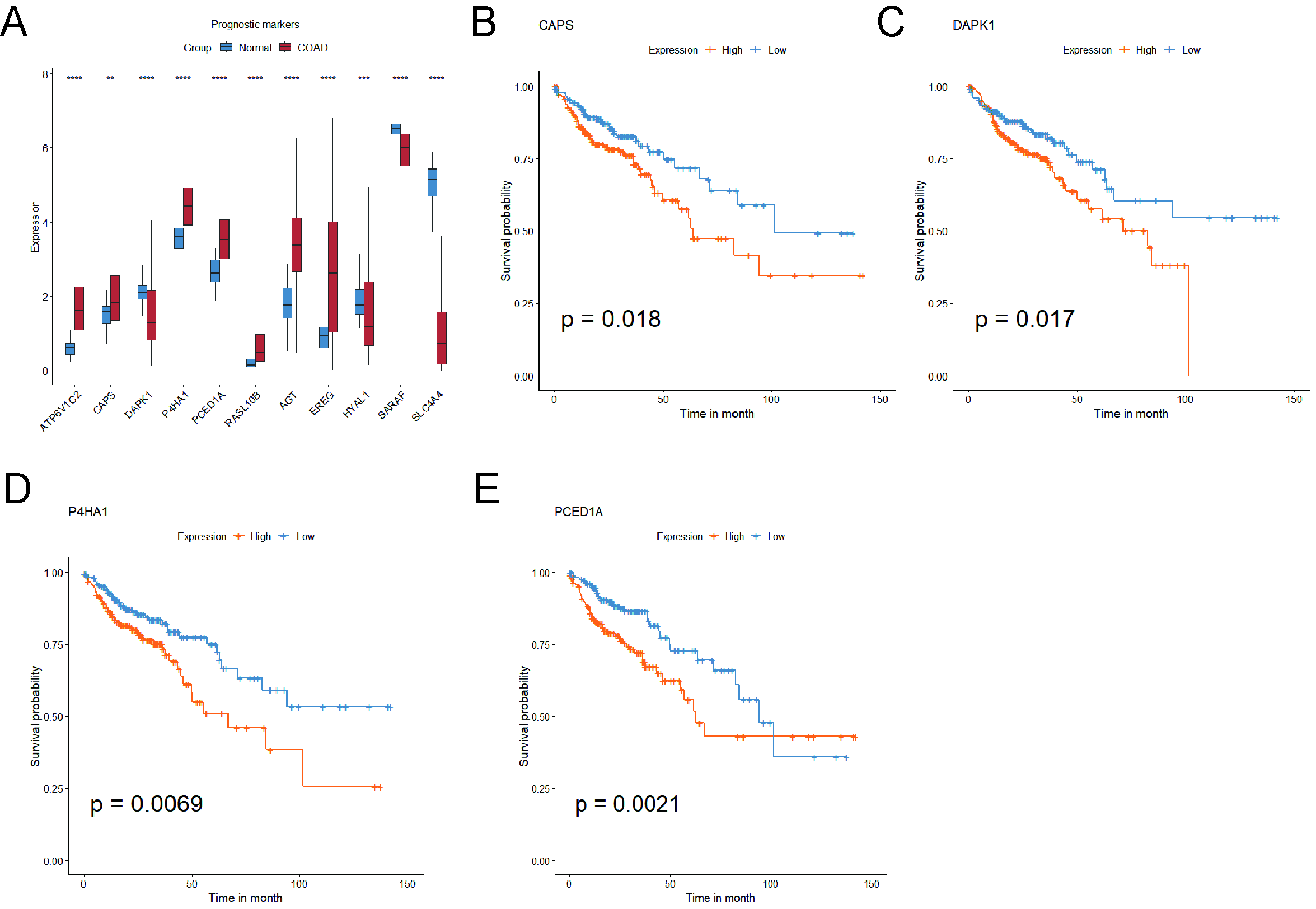

Supplement: Supplementary Figure 4 — Differential expression and prognostic significance of succinylation-related prognostic genes in CRC. (A) Differential expression analysis of 11 succinylation-related prognostic genes. (B) Kaplan-Meier overall survival (OS) curves for CAPS in CRC patients. (C) Kaplan-Meier OS curves for DAPK1 in CRC patients. (D) Kaplan-Meier OS curves for P4HA1 in CRC patients. (E) Kaplan-Meier OS curves for PCED1A in CRC patients. **p < 0.01; ***p < 0.001;***p < 0.0001. [file Image4.tif]
