# Supplementary material for: Succinylation-related molecular activities in cancer: metabolic adaptations, immune landscape, and prognostic significance in colorectal cancer
Source: Front Immunol. 2025 May 20;16:1571446. doi: 10.3389/fimmu.2025.1571446 (PMC12129993; doi:10.3389/fimmu.2025.1571446)
Supplement: Supplementary file 5 [file DataSheet1.pdf]

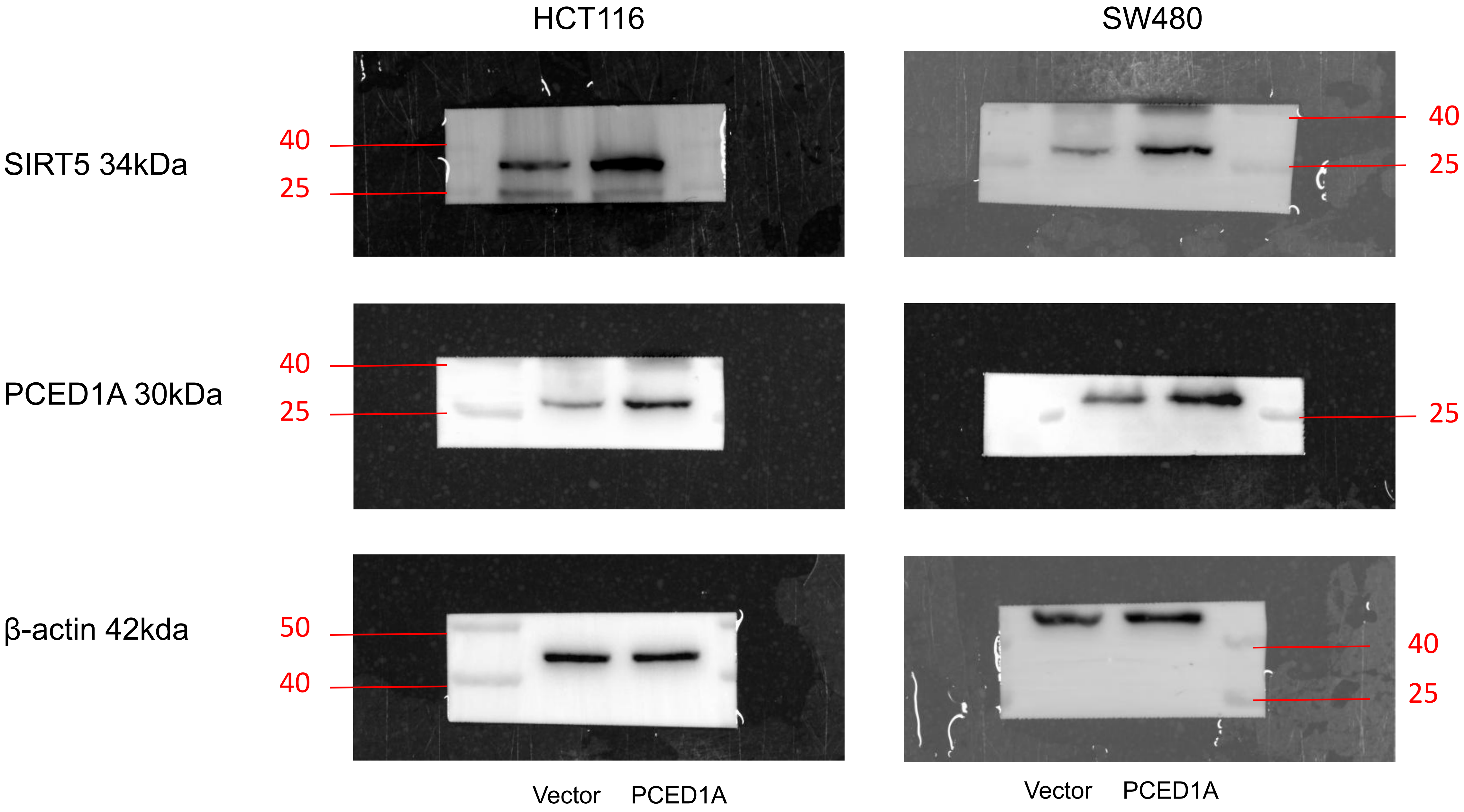
HCT116
SW480
SIRT5 34kDa
PCED1A 30kDa
β-actin 42kda
40
25
40
25
50
40
40
25
25
40
25
Vector
PCED1A
Vector
PCED1A

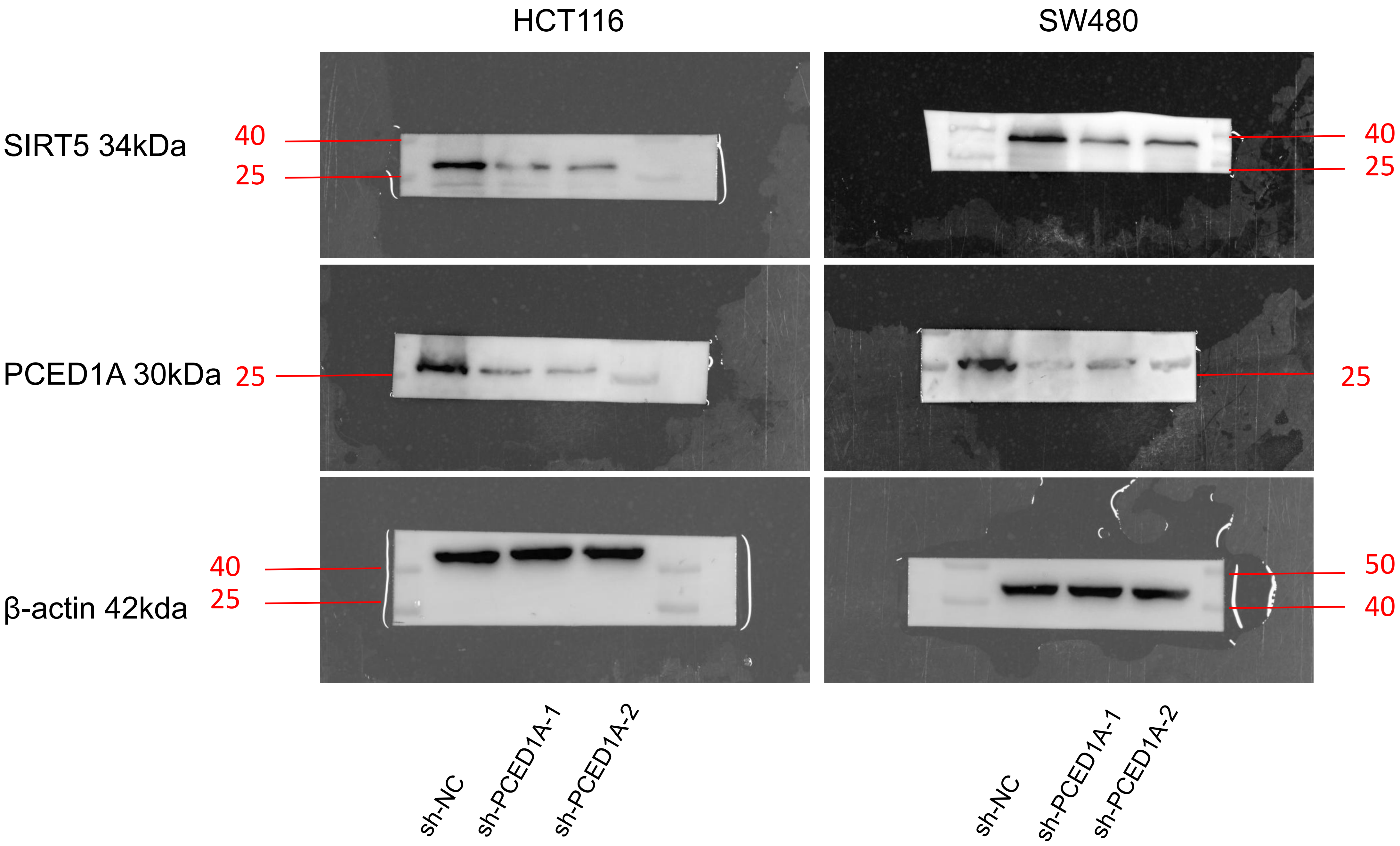

HCT116
SW480
SIRT5 34kDa
PCED1A 30kDa
β-actin 42kda
40
25
40
25
25
25
40
25
50
40
sh-NC
sh-PCED1A-1
sh-PCED1A-2
sh-NC
sh-PCED1A-1
sh-PCED1A-2

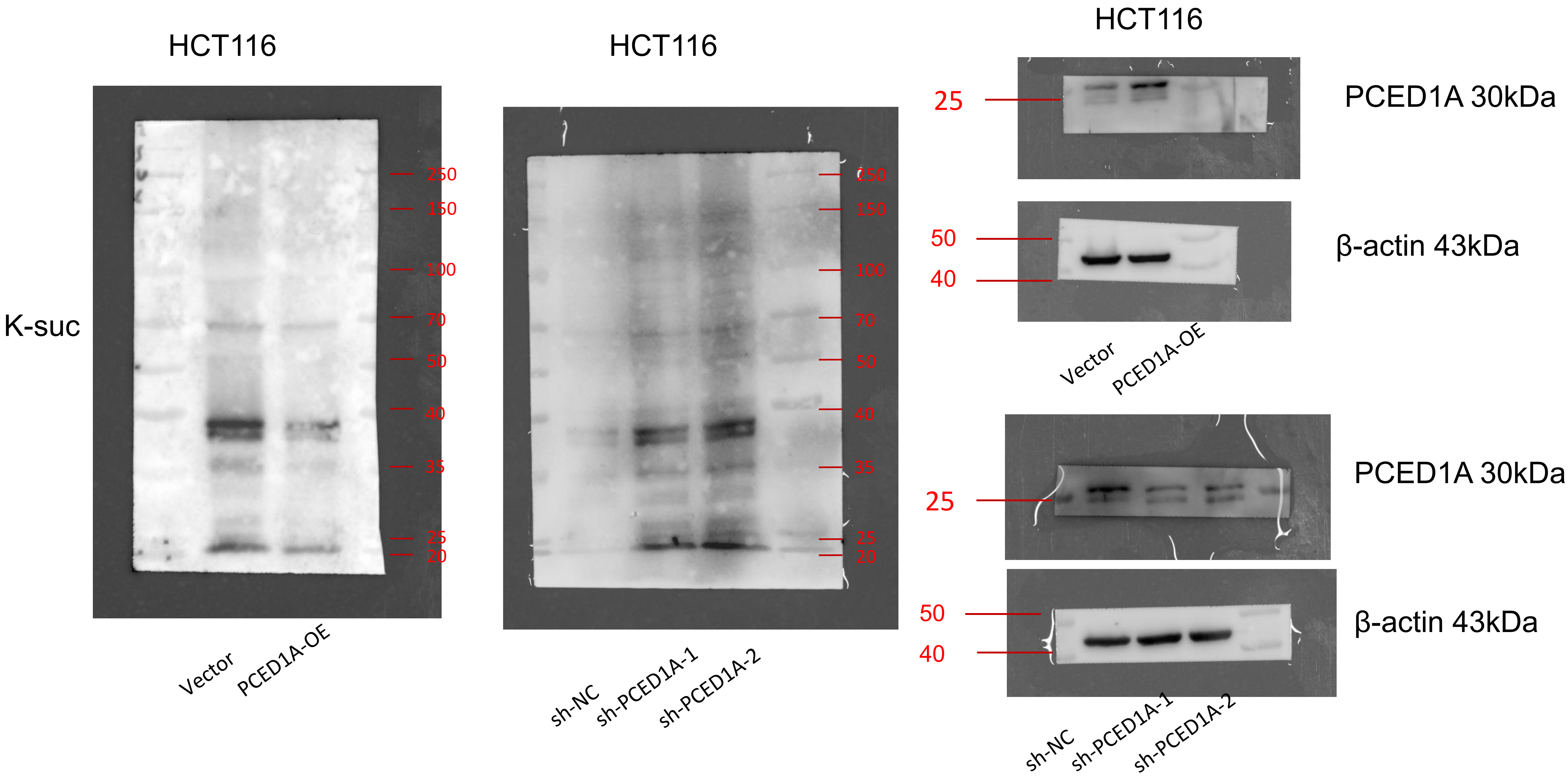
HCT116
K-suc
250
150
100
70
50
40
35
25
20
Vector
PCED1A-OE
HCT116
250
150
100
70
50
40
35
25
20
sh-NC
sh-PCED1A-1
sh-PCED1A-2
HCT116
25
PCED1A 30kDa
50
40
β-actin 43kDa
Vector
PCED1A-OE
25
PCED1A 30kDa
50
40
β-actin 43kDa
sh-NC
sh-PCED1A-1
sh-PCED1A-2
